# Rapid genetic screening with high quality factor metasurfaces

**Published:** 2021-10-15

**Authors:** Jack Hu, Fareeha Safir, John M. Abendroth, Jefferson Dixon, Benjamin A. Pinsky, Stefanie S. Jeffrey, Mark Lawrence, Jennifer A. Dionne

**Affiliations:** 1 Department of Materials Science and Engineering, Stanford University, 496 Lomita Mall, Stanford, CA 94305, USA; 2 Department of Mechanical Engineering, Stanford University, 440 Escondido Mall, Stanford, CA 94305, USA; 3 Laboratory for Solid State Physics, ETH Zürich, CH-8093 Zürich, Switzerland; 4 Department of Pathology, Stanford University School of Medicine, 300 Pasteur Drive, Stanford, CA 94305, USA; 5 Department of Medicine, Division of Infectious Diseases and Geographic Medicine, Stanford University School of Medicine, 300 Pasteur Drive, Stanford, CA 94305, USA; 6 Department of Surgery, Stanford University School of Medicine, 1201 Welch Road, Stanford, CA 94305, USA; 7 Department of Electrical & Systems Engineering, Washington University in St. Louis, 1 Brookings Drive, St. Louis, MO 63130, USA

## Abstract

Genetic analysis methods are foundational to advancing personalized and preventative medicine, accelerating disease diagnostics, and monitoring the health of organisms and ecosystems. Current nucleic acid technologies such as polymerase chain reaction (PCR), next-generation sequencing (NGS), and DNA microarrays rely on fluorescence and absorbance, necessitating sample amplification or replication and leading to increased processing time and cost. Here, we introduce a label-free genetic screening platform based on high quality (high-Q) factor silicon nanoantennas functionalized with monolayers of nucleic acid fragments. Each nanoantenna exhibits substantial electromagnetic field enhancements with sufficiently localized fields to ensure isolation from neighboring resonators, enabling dense biosensor integration. Quantitative detection of complementary target sequences via hybridization occurs simultaneously for arrays of sensing elements patterned at densities of 160,000 pixels per cm^2^. In physiological buffer, our nanoantennas exhibit average resonant quality factors of 2,200, allowing detection of purified SARS-CoV-2 envelope (E) and open reading frame 1b (ORF1b) gene fragments with high sensitivity and specificity (up to 94% and 96%) within 5 minutes of nucleic acid introduction. Combined with advances in nucleic acid extraction from complex samples (eg, mucus, blood, or wastewater), our work provides a foundation for rapid, compact, and high throughput multiplexed genetic screening assays spanning medical diagnostics to environmental monitoring.

Genetic screening methods have enabled significant advances in the prediction, detection, treatment, and monitoring of organism and ecosystem health. For example, respiratory panels identify pathogen nucleic acids indicative of infectious diseases like influenza and Coronavirus disease 2019 (COVID-19)[1, 2]; tissue and liquid biopsies detect cancerous genetic mutations, likelihood of recurrence, and are used to guide treatment[3, 4]; and emerging environmental DNA sensors monitor the health of oceans, freshwater, livestock, soil and air[5, 6]. Current genetic screening methods include polymerase chain reaction (PCR), next-generation sequencing (NGS), Sanger sequencing, and DNA microarrays. Each utilizes oligonucleotide amplification followed by optical tagging to sensitively detect target sequences. Despite their tremendous utility in laboratory settings, translation of these screening methods to clinical and point-of-care applications is ultimately limited by their reliance on “traditional” optical signal transduction (absorption and fluorescence). Even with the best optical tags, sensitive and specific readouts are generally only achieved with time consuming thermal cycling and/or costly reagents for nucleic acid amplification.

Rather than amplifying the concentration of the biomolecule, we postulated that light could be resonantly amplified to help enable compact, point-of-care biomarker screening methods. Photonic devices strongly confine and scatter light; when decorated with molecular probes, target analyte binding alters the optical signal due to subtle changes in the polarizability or refractive index of the resonator environment. Plasmonic sensors are among the most common affinity-based biosensors[7, 8, 9, 10, 11], but have larger limits of detection set by the metals’ intrinsic absorption; the resulting low quality factor (Q) resonances (Q~10) give rise to poor differentiation of small binding signals; the resonator’s sensing figure of merit (FOM), defined as sensitivity [resonant wavelength shift per refractive index unit (RIU) change] divided by the full width at half maximum (FWHM) of the mode, is *ca*. 1–10 RIU^−1^ for plasmonic sensors. More recently, dielectric nanoantennas and metasurface based sensors have been designed with Q factors of 10’s-100’s, with similar improvements in the FOM[12, 13, 14, 15, 16, 17, 18, 19]. Unlike high-Q whispering gallery mode resonators[20, 21, 22] and photonic crystal microcavity devices[23, 24], these metasurfaces can be illuminated from free space and far field scattering can be readily controlled, an advantage in the scalability and integration of sensors in imaging based devices.[25] However, these systems typically rely on delocalized resonant modes formed from extended two-dimensional arrays to improve Q factors and the resultant large modal volumes reduce responses to binding of small amounts of target molecules. Additionally, the larger footprint of these arrays limits the dense incorporation of sensing elements for multiplexed analyte detection and data driven analyses.

In this work, we report a new genetic analysis platform based on our lab’s development of high quality factor metasurfaces[26]. These metasurfaces consist of subwavelength nanoantennas that strongly confine light in the near field while affording precise control over far-field scattering. We design resonators that exhibit high average Q’s in buffered biological media of 2,200, with strong field penetration into the surrounding environment for sensitive biomarker detection. We show that the FOM of our sensors is 400 RIU^−1^, in good agreement with our computational model and significantly larger than existing nanophotonic sensors. We functionalize our resonators with self-assembled monolayers of DNA probes complementary to the SARS-CoV-2 E and ORF1b gene sequences. Hybridization of target nucleic acid fragments to the surface probes results in a rapid (<5 minute) change in the resonant wavelength, with sensitivities and specificities up to 94% and 96%, respectively. Due to the spatially localized nature of the high-Q resonances, individual sensing pixels can be patterned at densities of 160,000+ features per square cm, promising analyte parallelizability across a multitude of biomarkers.

## Individually addressable high-Q resonator sensing platform

**Fig. 1.**
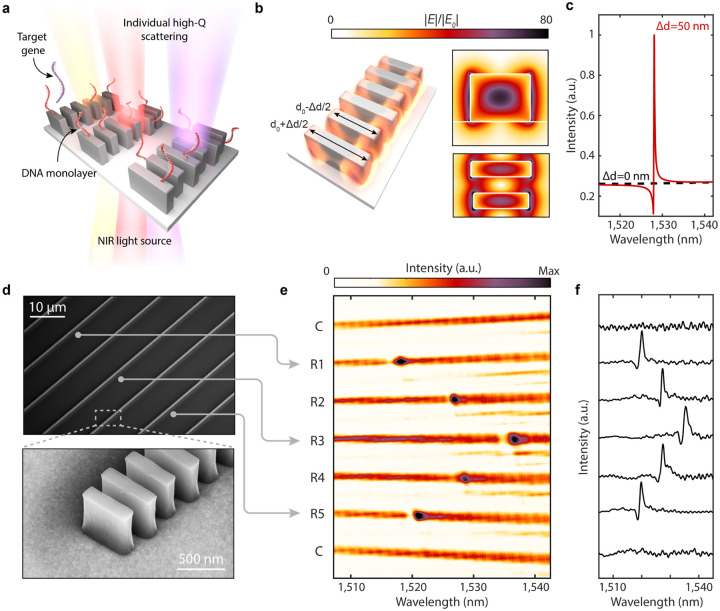
Design of high-Q sensors. **a**, Metasurface arrays of high-Q guided mode resonators consisting of perturbed chains of silicon blocks interfaced with DNA probes for targeted gene detection. Geometrical parameters of the resonators are height (h) = 500 nm, *d*_0_ = 600 nm, thickness (t) = 160 nm, block spacing (*a*_*y*_ = 330 nm), inter-chain spacing (*a*_*x*_ = 10 *μ*m), and Δd varied between 30–100 nm. **b**, Simulated electric near-field enhancements for resonator with Δd = 50 nm. **c**, Simulated cross-polarized transmission response of metasurface illuminated with normally incident linearly polarized plane waves. Responses normalized to intensity maximum of perturbed resonator. **d**, SEM micrographs of metasurface device composed of multiple individually monitored and tuned resonators. **e**, Spectral image from array with 7 resonators where C denotes nanostructures with no perturbation Δd = 0 nm and R1-R5 having perturbation Δd = 50nm. Resonance positions are modulated by adjusting block length where *d*_0_ = 595 nm for R1 & R5, *d*_0_ = 600 nm for R2 & R4, and *d*_0_ = 605 nm for R3. **f**, Row averaged transmitted intensities corresponding to **e**.

Figure 1a illustrates our sensor design, which consists of rows of silicon nanoblocks illuminated with near-infrared light. Each row constitutes a one-dimensional guided-mode resonant (GMR) metasurface; the periodic modulation of block widths within each row, characterized by Δd, allows for finite, but suppressed dipolar radiation and free space coupling to otherwise bound waveguide modes (Supplementary Note 1 and Supplementary Fig. 3)[26, 27, 28, 29]. The resulting long resonant lifetime translates to strong electric near-field enhancements (Fig. 1b). Notably, electric fields at the surface of Si blocks are enhanced by 80x. Due to the gaps between discrete silicon blocks within the resonator, 29% of the electric field energy is exposed to the surrounding medium compared with 8% in a continuous or partially notched waveguide (Supplementary Note 2 and Supplementary Fig. 4). This field concentration in the gaps leads to greater sensitivity to surface-bound analytes. Additionally, these silicon resonators exhibit sharp scattering responses in the far-field. As seen in Figure 1c, calculated transmission spectra Q-factors exceed 5,000 for Δd=50 nm, and can be further increased with decreased Δd (*vide infra*).

We fabricate silicon resonators atop a sapphire substrate (Fig. 1d) (see Methods). Utilizing a near-infrared supercontinuum laser and spectrometer equipped transmission microscope (Supplementary Fig. 1), we illuminate the metasurfaces at normal incidence and simultaneously measure the transmitted spectra from multiple resonators (Fig. 1e). By modulating the block lengths in adjacent nanostructures by ± 5 nm, we intentionally vary the spectral position of the resonant mode, highlighting that each waveguide structure can be individually addressed and tuned as a distinct resonator (Fig. 1e & 1f); in other words, our high-Q resonances do not rely on inter-chain coupling or an extended 2-D array effect. This spatial localization of the optical modes makes our platform ideally suited for the integration of densely distributed and multiplexed sensor arrays.

## Guided-mode resonant metasurface characterization

**Fig. 2.**
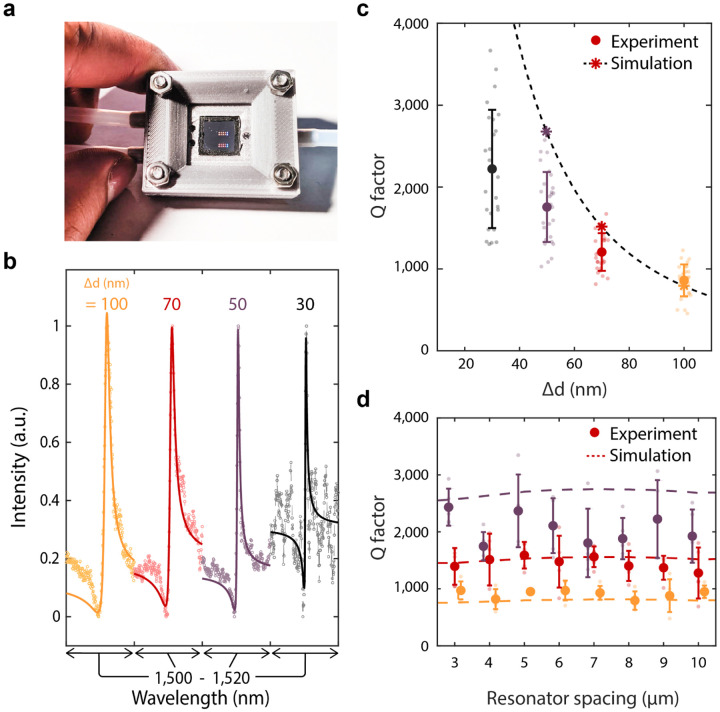
Fluid cell characterization of metasurfaces. **a**, Photo of metasurface chip enclosed in fluid cell. **b**, Spectra from resonators with varying Δd. Solid lines represent fits to a Lorentzian oscillator. **c**, Quality factor of resonances with different Δd. Bold markers and error bars are the mean and standard deviation for N=30 resonators at each condition. Stars represent simulated values and the dashed line is a fit to predicted values from coupled mode theory (Supplementary Note 3). **d**, Quality factor as a function resonator spacing where mean and standard deviation are for N=5 resonators at each condition.

Our metasurfaces are sealed in a 3-D printed fluid cell (Fig. 2a) and characterized in phosphate-buffered saline (PBS) solution (1x concentration) to represent physiological conditions for biomolecule detection. In Fig. 2b, we vary the perturbation Δd along the block chain from Δd = 100 nm to Δd = 30 nm and observe a decrease in the resonant linewidth for 25–30 individual resonators at each condition (Fig. 2b & 2c). Importantly, in our high-Q metasurface design, the coupling strength between free space radiation and the GMR is dictated by the degree of asymmetry along the waveguide. Since silicon is lossless in the near infrared, radiative loss dominates the GMR resonant lifetime and Q factor. Thus, shrinking Δd we observe scattering responses with average Q factors of 800 (at Δd = 100 nm) increasing to 2,200 at Δd= 30 nm and even observing Q’s above 3,000 for individual resonators (Fig. 2c). These Q factors represent a two to three order of magnitude increase compared to reported plasmonic biosensors, and a significant (>5–10x) increase compared to other metasurface biosensors[16, 17, 19, 30, 31], yielding a FOM of ~400 (Supplementary Fig. 7). Our experimental Q factors are slightly lower than numerically predicted and are likely limited due to scattering losses caused by fabrication imperfections. We also note that water has non-negligible absorption in the 1,500 nm wavelength range that may limit our attainable experimental Q factors (Supplementary Note 3 and Supplementary Fig. 5). Designing future metasurface resonances in an optical transparency window of biological media (such as 1,300 nm) and optimizing fabrication processes may further improve performance, with Q factors in the millions potentially attainable; future iterations of our metasurface could offer the single particle sensitivity of high-Q microcavities,[20, 22] but with the ease of integration and compactness afforded by free-space coupling.

Due to the localization of the mode along each individual chain, resonators can be spaced laterally at least as close as 3 *μ*m without affecting the GMR (Fig 2d). Based on our fabricated waveguide length of 200 *μ*m, our devices feature sensor arrays with densities of over 160,000 sensors per cm^2^. Due to the slow group velocities of the GMR’s, losses due to finite size effects can be suppressed[29, 32], and 50 *μ*m waveguides can be fabricated with comparable Q (Supplementary Fig. 6), yielding feature densities over 600,000 sensors per cm^2^. These large sensor densities offer an avenue for robust statistical analysis in diagnostic studies as well as a platform for multiplexed detection of many distinct biomarkers in parallel.

## Self-assembled monolayer functionalization and sensing

**Fig. 3.**
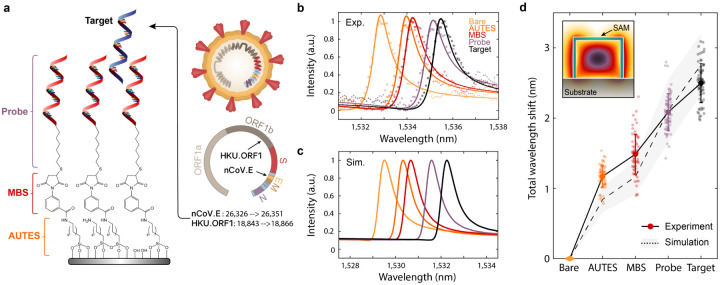
DNA monolayer functionalization and resonant wavelength shift measurement. **a**, Schematic of chemical components utilized in immobilizing DNA self-assembled monolayers (SAM) onto the silicon nanostructures. Target DNA fragments for this study are portions of the E and ORF1b genes from the SARS-CoV-2 virus. **b**, Experimentally measured and **c**, simulated resonance wavelength shift responses with the addition of each molecular layer in the SAM, including complementary nCoV.E target binding. Markers in **b**, correspond to measured data points while solid lines show fits to a Lorentzian oscillator. The difference in absolute wavelength values between experimental and simulated spectra can be attributed to slight dimension variations in the fabricated structures. **d**, Total resonant wavelength shift during SAM functionalization and DNA sensing as referenced from initial measurements on bare silicon structures. Markers represent individual measurements from N=75 independent resonator devices and bolded markers and error bars are the mean and standard deviation of the measurements.

To utilize our sensor arrays for gene detection, we modified the silicon surface with DNA monolayers, where complementary nucleic acid sequences serve as capture molecules for a specified target. Self-assembled monolayers (SAMs) are deposited in a three-step process to covalently link 26 base pair single-stranded DNA (ssDNA) probes over the entire metasurface chip surface. The silicon surface is first functionalized with an amine-terminated silane (11-aminoundecyltriethoxysilane, AUTES), and then cross-linked via a heterobifunctional molecule (3-maleimidobenzoic acid N-hydroxysuccinimide ester, MBS) to thiolated ssDNA probes (Methods and Supplementary Fig. 2). In this study, we considered nucleic acid fragment targets of the envelope (E) and open reading frame 1b (ORF1b) genes of the SARS-CoV-2 virus (GenBank accession: MT123293.2 positions 26326→ 26351 and 18843→ 18866, respectively, also see Supplementary Table 1)[33, 34](Fig. 3a). As a proof of principle, we use synthetic DNA targets, but note that viral RNA will analogously hybridize to complementary DNA probes[35, 36].

In Fig. 3b, measured spectra show clear resonant wavelength shifts as consecutive molecular monolayers of AUTES, MBS, and the probe DNA are grafted to the resonator surface. Monolayers were modeled as thin dielectric shells surrounding the silicon blocks and simulated responses show close agreement with the experimental resonance shifts (Fig. 3c)(Supplementary Note 6 and Supplementary Fig. 8). Upon adding the target SARS-CoV-2 gene, a clear, 0.4nm resonant shift is observed (Fig 3d). Data was collected from N=75 individual resonators or chains of silicon blocks, and we note that the high density of sensing elements on our chips can enable significant increases in measurement throughput compared to typical photonic sensors where signals are averaged over larger 2-D arrays. The deviation between experimental and simulated wavelength shifts for the AUTES and MBS layers is likely due to the tendency for aminosilane molecules to form multi-layer structures; differences in the attachment of DNA probes and subsequent target hybridization are likely due to a strong influence of steric hindrance and electrostatic repulsion effects on the packing density and hybridization efficiency of the DNA strands[37, 38, 39, 40].

## Rapid and specific gene fragment detection

**Fig. 4.**
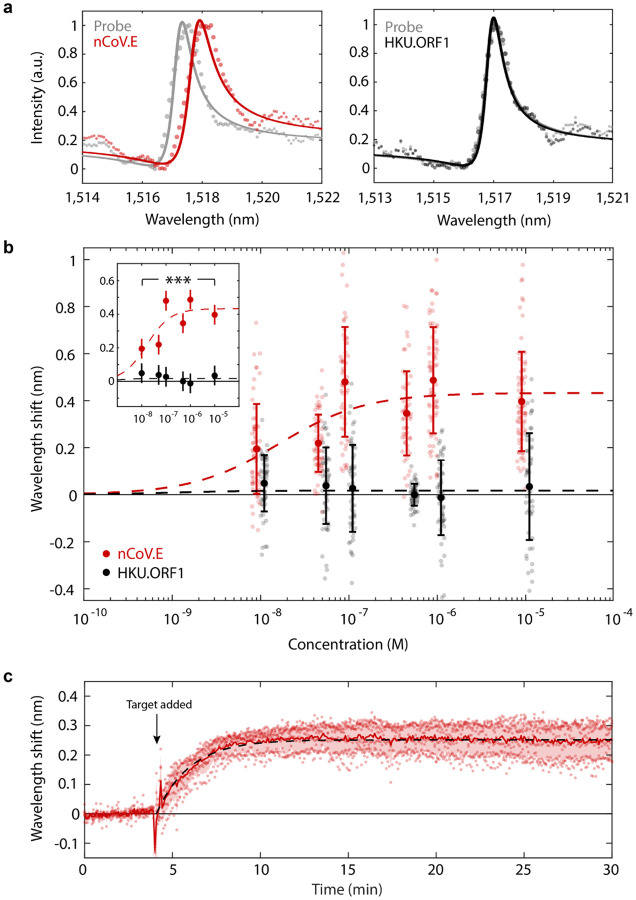
Biosensing demonstration with SARS-CoV-2 gene fragment targets. **a**, Measured spectra from individual resonators indicate significant wavelength shifts with complementary DNA binding and minimal signal changes when introduced to non-complementary sequences. **b**, Concentration dependent binding responses for both nCoV.E and HKU.ORF1 targets incubated on metasurface devices functionalized with only nCoV.E complementary probes. Error bars indicate standard deviations of measurements from N= 50–75 measurements from distinct resonators for each target and concentration condition. Dashed lines show fits to the Hill equation (Supplementary Note 8). (Inset) Two-way ANOVA and post-hoc Tukey’s HSD tests confirm statistically significant differences in binding responses for nCoV.E and HKU.ORF1 targets (Supplementary Table 3). Markers represent mean values and bars represent 99% confidence intervals. ***P<0.002 vs. non-complementary targets. **c**, Kinetic binding responses for 6 resonators incubated with 100 nm of nCoV.E targets. Solid red line is the mean of experimental measurements and the dashed line is a fit to the Langmuir adsorption model (Supplementary Note 8).

Pairing our resonators with specific probe DNA sequences offers specificity in target gene detection. To confirm specificity, we modify target DNA strands with ATTO590 fluorescent labels and incubate sensors functionalized with probes that are only complementary to the nCoV.E sequence. Fluorescence imaging of sensors exposed to 10 *μ*M solutions of target nCoV.E and HKU.ORF1 show significant binding only for the complementary E gene target and minimal signal for the non-complementary ORF1 strands (Supplementary Fig. 10). This target specificity is also measured in the resonator scattering spectra, where resonance wavelength shifts are significant for complementary target-probe conditions and suppressed for non-specific binding (Fig. 4a).

Our sensors exhibit concentration dependent responses from 1 *μ*M to 10 nM (Fig. 4b). Measurements are taken for N=50–75 individual resonators at each target and concentration condition. The large variability in resonant wavelength shifts at each concentration are likely due to the stochastic nature of surface binding; notably, the signal from any particular resonator will depend on the local concentration and spatial position of bound targets (and hence binding at sites with the greatest electric field concentration will produce larger resonant shifts). Additionally, signal variation is also likely introduced through the hydrolytic degradation of silane layers in aqueous solutions during functionalization and hybridization experiments[37, 38] (as seen in blue-shifted data points in Fig. 4b). We expect optimization of the surface functionalization homogeneity and stability to dramatically improve the performance of our sensors. Importantly, we are confident that this background signal, which is currently the dominant factor limiting our resolution, comes entirely from the instability of the sensing environment and not the photonic resonators themselves. We ultimately expect our detection threshold to be limited by the resonant linewidth, with shifts <0.1*FWHM easily measurable. We note that on our metasurface chips the silane linking chemistry will also non-specifically functionalize DNA probes to the surface oxide of the sapphire substrate. We estimate that only 0.0003% of surface bound target molecules contribute to the resonance shift of each resonator. Thus, even with the current background signal, our limit of detection could be reduced from 10 nM down to 10 fM with the introduction of microfluidic channels where only resonator regions are exposed to target molecules, utilization of a silicon specific surface functionalization process, or incorporation of additional nanostructures to isolate resonators from one another and increase sensor densities further[41]. Additionally, the concentration dependent range of our device can potentially be tuned to different values of analyte concentration through modification of surface probe densities[42].

Two-way analysis of variance (ANOVA) and post-hoc Tukey’s range test indicates that differences in scattered shift signals were statistically different for complementary vs. non- complementary targets at all tested concentrations (Fig. 4b inset)(Supplementary Tables 2 and 3). The increased measurement throughput and larger sample sizing of our platform can be used to significantly improve the accuracy of diagnostic studies, where multiple measurement redundancies allow for improved quantification and classification of sample populations. For example, we can classify “positive” complementary target detection against “negative” non-complementary target detection at each concentration based on thresholding resonant wavelength shift signals. Varying the threshold signal produces a receiver operating characteristic (ROC) curve, and the positive and negative signal discrimination is quantified as the area under the ROC curve (AUC). From this analysis, our sensors exhibit AUC values up to 0.98 (where AUC = 1 indicates perfect signal discrimination and 0.5 represents no discrimination) and high sensitivity and specificity of 94 and 96% respectively (Supplementary Fig. 11). This increased digitization of target gene binding may also be paired with machine learning based analysis for further improved accuracy or to allow for discrimination of small signals due to genetic variants and point mutations[43].

Real-time measurement of resonators shows rapid target binding responses for a 100 nM solution of nCoV.E complementary targets measured across six representative resonators (Fig. 4c). Changes in the resonant wavelength greater than the measurement noise are detected within seconds and the binding signal plateaus within 5 minutes of sample introduction. The signal response shows excellent agreement with the Langmuir adsorption model (dashed line Fig. 4c) with an observed hybridization rate constant of 7×10^−3^ s^−1^, comparable to other hybridization capture assays[44, 45]. These fast binding kinetics highlight a key advantage of chip-based approaches over conventional detection techniques that require time-intensive sequence amplification cycles.

## Conclusions

Our nanophotonic device offers a new platform for high throughput molecular analysis. We have demonstrated free space illuminated resonators with high-Q resonances in physiological media (2,200+) that can be patterned, tuned, and measured at densities exceeding 160,000 pixels per cm^2^. Even larger Q’s and greater feature densities are attainable in our platform with improved fabrication processes to reduce scattering losses from structural inhomogeneities, reduced absorption losses from biological media, and inclusion of photonic mirror elements to suppress light leakage as resonator chains are truncated below 50 *μ*m. Interfaced with DNA probes, our metasurface design enables rapid, label-free, and highly digitized genetic screening that can bridge many of the challenges faced by conventional genetic analysis techniques. Paired with bioprinting procedures where different gene sequence probes are spotted across distinct sensing pixels, our high-Q metasurface chips can provide the foundation for rapid, label-free, and massively multiplexed photonic DNA microarrays. Furthermore, our nanophotonic chips are amenable to intensity imaging and/or hyperspectral imaging techniques that provide signal binding information without the need for a spectrometer[15, 30], further reducing complexity and costs towards point of care genetic screening. Our platform promises unique possibilities for widely scaled and frequently administered genetic screening for the future of precision medicine, sustainable agriculture, and environmental resilience.

## Methods

### Computational design

Electromagnetic simulations were performed with the Lumerical FDTD Solver. Metasurfaces were simulated with periodic boundary conditions in the x and y directions and perfectly matched layer (PML) boundary conditions in the z direction. Structures were excited with a plane wave polarized at 45° and injected from the negative z direction through a sapphire substrate. Transmission spectra were computed using a power monitor placed in the far field of the metasurface in the +z direction. Cross polarized transmission intensity was calculated as Power(−45°)/(Power(−45°)+Power(+45°)).

### Fabrication

The metasurfaces were fabricated using standard lithographic procedures. First, 500 nm, single crystal silicon-on-sapphire (MTI Corp.) substrates were cleaned via sonication in acetone and isopropyl alcohol. The substrates were baked at 180 °C before spin coating with hydrogen silsesquioxane (HSQ) negative tone resist (XR-1541–06, Corning). The resist was baked for 40 min at 80 °C. To reduce charging, a charge dissipation layer (e-spacer, Showa Denko) was spin coated over the HSQ resist and baked again for 5 min at 80 °C. The metasurface patterns were defined by a 100 keV electron beam in a JEOL JBX-6300FS EBL system. Patterns were developed for 120 seconds in a 25% solution of tetramethylammonium hydroxide. Reactive ion etching with Cl2, HBr, and O2 chemistries were utilized to transfer the pattern to the silicon layer (Lam TCP 9400). The HSQ resist was removed using 2% hydrofluoric acid in water and the samples were then cleaned using a Piranha solution (9:1 H_2_SO_4_:H_2_O_2_) heated to 120 °C. The silicon nanostructures were passivated by heating for 30 min at 800 °C in a furnace to grow a ~ 4 nm oxide layer.

### Optical characterization

Resonator spectra were measured in a home-built near-infrared microscope shown in Supplementary Figure 1. Samples were illuminated via a broadband NKT supercontinuum laser with a collimated fiber output. A polarizer P1 was set to create linearly polarized incident illumination at a 45° angle with respect to the metasurface structures. The beam is weakly focused onto the sample through the sapphire substrate at normal incidence with a lens L2 (f= 50 mm) to an approximate spot size of 200 *μ*m. Additionally, all optical measurements in this work were taken with sample chips sealed in a fluid cell and immersed in PBS 1X. The scattered light is collected through a 50X objective lens (Olympus LCPLN50XIR) and directed through a cross-polarized polarizer P2 at −45° to reduce the substrate Fabry-Perot signal. The scattered light is then focused via a lens L3 (f=75 mm) into a SPR-2300 spectrometer (Princeton Instruments). The broadband signal is diffracted via a diffraction grating (600 g/mm, blase wavelength 600 nm, Princeton Instruments) and focused onto an air-cooled InGaAs detector (NiRvana, Princeton Instruments). All spectral measurements are collected as the average of three successive 200 millisecond acquisitions. Spectral features were analyzed by fitting the data with the function:

$$T={\left|a_{r}+a_{i}i+\frac{b}{f-f_{0}+\gamma i}\right|}^{2}$$

where *T* is the scattered intensity from a superposition between a constant complex background, *a*_*r*_ + *a*_*i*_*i*, and a Lorentzian oscillator with resonant frequency *f*_0_ and full-width at half-maximum of 2*γ*. The quality factor is then calculated as *Q* = *f*_0_/2*γ*.

### Surface functionalization

Self-assembled monolayers of single stranded probe DNA was interfaced to the silicon metasurfaces through a multi-step chemical functionalization process summarized in Supplementary Figure 2. To activate the silicon surface for functionalization, the samples were immersed in a Piranha solution (9:1 H_2_SO_4_:H_2_O_2_) heated to 120 °C for 20 min to hydroxylate the surfaces. Next, samples were immersed in a 0.1 mM solution of 11-aminoundecyltriethoxysilane (Gelest Inc.) in ethanol, sealed, and left for overnight for 18–24 hrs. The samples were rinsed in fresh ethanol for 5 min (3X) and then baked for 1 hr at 150 °C to form a stable silane layer. A hetero-bifunctional cross linking molecule was attached to the silane layer through immersion in a 1mM solution of 3-maleimidobenzoic acid N-hydroxysuccinimide ester (Millipore Sigma) dissolved in a 1:9 (v/v) mixture of dimethyl sulfoxide and PBS for 1 hr. Samples were then rinsed thoroughly with deionized water and blown dry with N_2_ gas. Single stranded DNA probes were obtained from Integrated DNA Technologies (Coralville, IA) modified with a disulfide tether on the 5’ ends. The as received DNA probes were disperesed in 50 *μ*L of tris-EDTA buffer, pH 8.0, and mixed with 30 mg of DL-dithiothreitol for at least 1 hr to reduce the disulfide moieties to thiols. The probes were then purified via gravity-flow size exclusion chromatography using illustra NAP-5 columns. The concentration of the eluted DNA solutions were determined using UV absorption signatures (Varian Cary 500 UV-Vis Spectrophotometer). For the functionalization reaction, portion of the stock solution were then diluted to 20 *μ*M in PBS 1x with added divalent cations of 100 mM MgCl_2_. The DNA probe solution was pipetted onto each sample and incubated overnight (~18–24 hrs) in a dark and humid environment. Samples were rinsed with PBS 1X and then soaked in a PBS solution with added salt to a concentration of 1M NaCl for 4 hours to remove any loosely bound or physiosorbed oligonucleotides. Samples were then rinsed with PBS 1X and deionized water and dried with N_2_ gas. Samples corresponding to optical measurements in main text Fig. 3 were measured before and after each functionalization step with additional deionized water rinsing and N_2_ drying before the next chemical processing step. Samples corresponding to main text Fig. 4 were optically characterized only before and after target DNA hybridization.

### DNA hybridization

For static DNA hybridization measurements (all presented data in main text excluding Fig. 4c), a baseline spectroscopic measurement was taken on metasurfaces that had been functionalized with a probe DNA monolayer. Probes with sequences corresponding to the E gene of the SARS-CoV-2 virus were used in all experiments. Following baseline measurements, samples were rinsed with DI water and dried. A target DNA solution corresponding to either complementary E gene or non-complementary ORF1b gene fragments (Supplementary Table 1) was produced by diluting a 100 *μ*M stock solution to the desired concentration in 1X PBS. Additional divalent cations corresponding to 100 mM MgCl_2_ were added to the solution to increase hybridization efficiency and speed. A 100 *μ*L droplet of the target solution is then pipetted onto each sample chip and incubated for 30 min in a dark environment. Samples are rinsed in PBS 1X and deionized water before final optical characterization.

For dynamic DNA hybridization measurements presented in Fig. 4c of the main text, samples functionalized with DNA probes were placed in a fluid cell and mounted in the optical transmission set up described above. Spectral acquisitions were collected at 10 second intervals, and baseline measurements of the metasurfaces immersed in a pure hybridization solution with no nucleic acids were taken for 4 minutes. Next, excess volume of the target solution containing nucleic acids was flowed into the fluid cell from a syringe for 10 seconds via inlet tubing to displace the pure hybridization solution and completely fill the cell with target solution. Spectra were monitored for an additional 20 minutes and wavelength shifts were calculated based on changes compared to the average resonance wavelength obtained from the initial 4 minute baseline measurement.

## Supplementary Material

1
