# Rapid genetic screening with high quality factor metasurfaces

**Published:** 2021-10-15

**Authors:** Jack Hu, Fareeha Safir, Kai Chang, Sahil Dagli, Halleh B. Balch, John M. Abendroth, Jefferson Dixon, Parivash Moradifar, Varun Dolia, Malaya K. Sahoo, Benjamin A. Pinsky, Stefanie S. Jeffrey, Mark Lawrence, Jennifer A. Dionne

**Affiliations:** 1 Department of Materials Science and Engineering, Stanford University, 496 Lomita Mall, Stanford, CA 94305, USA; 2 Department of Mechanical Engineering, Stanford University, 440 Escondido Mall, Stanford, CA 94305, USA; 3 Department of Electrical Engineering, Stanford University, 350 Jane Stanford Way, Stanford, CA 94305, USA; 4 Laboratory for Solid State Physics, ETH Zürich, CH-8093 Zürich, Switzerland; 5 Department of Pathology, Stanford University School of Medicine, 300 Pasteur Drive, Stanford, CA 94305, USA; 6 Department of Medicine, Division of Infectious Diseases and Geographic Medicine, Stanford University School of Medicine, 300 Pasteur Drive, Stanford, CA 94305, USA; 7 Department of Surgery, Stanford University School of Medicine, 1201 Welch Road, Stanford, CA 94305, USA; 8 Department of Electrical & Systems Engineering, Washington University in St. Louis, 1 Brookings Drive, St. Louis, MO 63130, USA

## Abstract

Genetic analysis methods are foundational to advancing personalized and preventative medicine, accelerating disease diagnostics, and monitoring the health of organisms and ecosystems. Current nucleic acid technologies such as polymerase chain reaction (PCR), next-generation sequencing (NGS), and DNA microarrays rely on fluorescence and absorbance, necessitating sample amplification or replication and leading to increased processing time and cost. Here, we introduce a label-free genetic screening platform based on high quality (high-Q) factor silicon nanoantennas functionalized with monolayers of nucleic acid fragments. Each nanoantenna exhibits substantial electromagnetic field enhancements with sufficiently localized fields to ensure isolation from neighboring resonators, enabling dense biosensor integration. We quantitatively detect complementary target sequences using DNA hybridization simultaneously for arrays of sensing elements patterned at densities of 160,000 pixels per cm^2^. In physiological buffer, our nanoantennas exhibit average resonant quality factors of 2,200, allowing detection of two gene fragments, SARS-CoV-2 envelope (E) and open reading frame 1b (ORF1b), down to femtomolar concentrations. We also demonstrate high specificity sensing in clinical nasopharyngeal eluates within 5 minutes of sample introduction. Combined with advances in biomarker isolation from complex samples (e.g., mucus, blood, wastewater), our work provides a foundation for rapid, compact, amplification-free and high throughput multiplexed genetic screening assays spanning medical diagnostics to environmental monitoring.

Genetic screening methods have enabled significant advances in the prediction, detection, treatment, and monitoring of organism and ecosystem health. For example, respiratory panels identify pathogen nucleic acids indicative of infectious diseases like influenza and Coronavirus disease 2019 (COVID-19)[1, 2]; tissue and liquid biopsies detect cancerous genetic mutations and likelihood of recurrence, and are used to guide treatment[3, 4]; and emerging environmental DNA sensors monitor the health of oceans, freshwater, livestock, soil and air[5, 6]. Current genetic screening methods include polymerase chain reaction (PCR), next-generation sequencing (NGS), Sanger sequencing, and DNA microarrays. Each utilizes oligonucleotide amplification followed by optical tagging to sensitively detect target sequences. Despite their tremendous utility in laboratory settings, translation of these screening methods to clinical and point-of-care applications is ultimately limited by their reliance on “traditional” optical signal transduction (absorption and fluorescence). Even with the best optical tags, sensitive and specific readouts are generally only achieved with time consuming thermal cycling and/or costly reagents for nucleic acid amplification.

Nanotechnology-based biosensors have promised new platforms for rapid and scalable bio-molecule detection without requiring biochemical amplification or target labeling. Miniaturized electronic and optical devices offer increased sensitivity due to their nanoscale control of electric and magnetic fields, as well as the potential for scalable multiplexing, owing to their compatibility with complementary metal-oxide-semiconductor (CMOS) fabrication processes. For example, field-effect transistor (FET) biosensors measure surface potential changes due to molecular binding events [7, 8, 9], while molecular tunnel junction sensors measure changes in tunneling current[10, 11, 12]. These sensors achieve ultra-high sensitivities with femtomolar detection limits, but reliably measuring samples in physiologically-relevant ionic media remains a challenge.

Complementing electronic sensors, photonic sensors promise high parallelization with more robust read-outs in realistic samples. Rather than amplifying or replicating the biomarker, photonic devices strongly confine and amplify the electromagnetic fields; when decorated with molecular probes, target analyte binding alters the optical signal due to subtle changes in the polarizability or refractive index of the resonator environment. The resonator’s sensing figure of merit (FOM) is generally defined as sensitivity (resonant wavelength shift per refractive index unit (RIU) change) divided by the full width at half maximum (FWHM) of the mode. Plasmonic sensors are among the most common affinity-based biosensors.[13, 14, 15, 16, 17] These metallic structures exhibit small mode volumes and dipole-like scattering, but generally only achieve FOM values of ca. 1–10 RIU^−1^, due to low quality factors (Q) that are limited by the metals’ intrinsic absorption (Q~10). Furthermore, due to absorption, these metallic-based structures can lead to sample heating that can degrade biomolecules.

More recently, metasurface based sensors have been designed with Q factors of 10’s-100’s, with similar improvements in the FOM[18, 19, 20, 21, 22, 23, 24, 25, 26, 27, 28, 29]. Unlike high-Q whispering gallery mode resonators[30, 31, 32] and photonic crystal microcavity devices[33, 34], these metasurfaces can be illuminated from free space and far field scattering can be readily controlled, an advantage to the scalability and integration of sensors in imaging based devices.[35] However, these systems typically rely on improving Q-factors using delocalized resonant modes formed from extended two-dimensional arrays; the resultant large modal volumes reduce responses to binding of small numbers of target molecules. Additionally, the larger footprint of these arrays (>100 by 100 um^2^) limits the dense incorporation of sensing elements for multiplexed analyte detection and data driven analyses.

In this work, we report a new genetic analysis platform based on our lab’s development of high quality factor metasurfaces[36]. These metasurfaces consist of subwavelength nanoantennas that strongly confine light in the near field while affording precise control over the far-field scattering. We design resonators that exhibit high average Q’s of 2,200 in buffered biological media, with strong field penetration into the surrounding environment for sensitive biomarker detection. Due to the spatial localization of the high-Q resonances, individual sensing pixels can be patterned at densities exceeding 160,000 features per cm^2^, promising detection parallelizability across a multitude of biomarkers. We functionalize our resonators with self-assembled monolayers of DNA probes complementary to the SARS-CoV-2 E and ORF1b gene sequences. Our sensors rapidly and sensitively detect 22-mer gene fragments within 5 minutes of sample introduction from micromolar to femtomolar concentrations. We further demonstrate high-specificity molecular screening in clinical nasopharyngeal eluates, validating the clinical applicability of our sensor platform for rapid, sensitive, and specific detection of target genes.

## Individually addressable high-Q resonator sensing platform

**Fig. 1.**
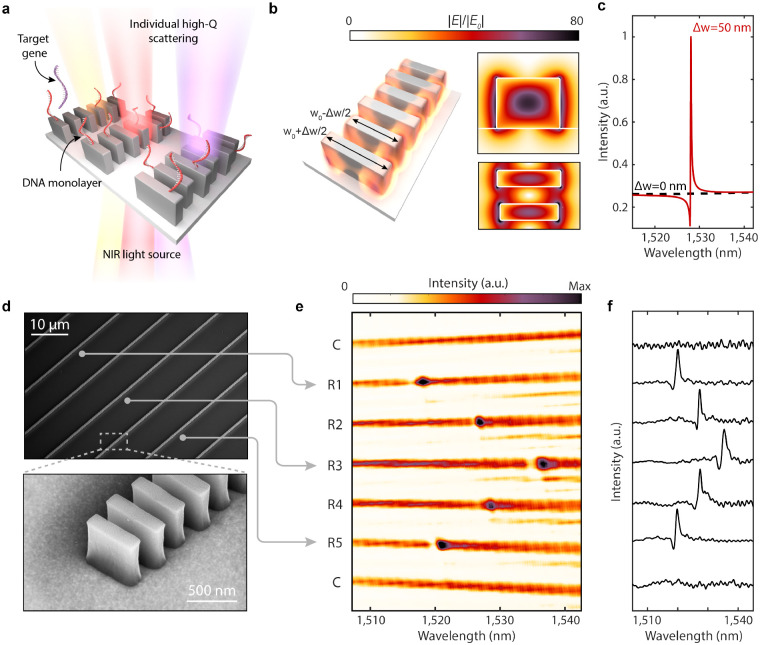
Design of high-Q sensors. **a,** Metasurface arrays of high-Q guided mode resonators consisting of perturbed chains of silicon blocks interfaced with DNA probes for targeted gene detection. Geometrical parameters of the resonators are height (h) = 500 nm, *w*_0_ = 600 nm, thickness (t) = 160 nm, block spacing (*a*_*y*_ = 330 nm), inter-chain spacing (*a*_*x*_ = 10 *μ*m), and Δw varied between 30–100 nm. **b**, Simulated electric near-field enhancements for a resonator with Δw = 50 nm. **c**, Simulated cross-polarized transmission response of metasurface illuminated with normally incident linearly polarized plane waves. Responses normalized to intensity maximum of perturbed resonator. **d**, SEM micrographs of metasurface device composed of multiple individually monitored and tuned resonators. **e**, Spectral image from array with 7 resonators where C denotes nanostructures with no perturbation Δw = 0 nm and R1-R5 having perturbation Δw = 50nm. Resonance positions are modulated by adjusting block length where *w*_0_ = 595 nm for R1 & R5, *w*_0_ = 600 nm for R2 & R4, and *w*_0_ = 605 nm for R3 to form the observed chevron pattern. **f**, Row averaged transmitted intensities corresponding to **e**.

Figure 1a illustrates our sensor design, which consists of closely-spaced silicon nanoblocks illuminated with near-infrared light. Each set of blocks constitutes a one-dimensional guided-mode resonant (GMR) nanoantenna; the periodic modulation of block widths, characterized by Δw, allows for finite, but suppressed dipolar radiation and free space coupling to otherwise bound waveguide modes (Supplementary Note 1 and Supplementary Fig. 3)[36, 37, 38, 39]. The resulting long resonant lifetime translates to strong electric near-field enhancements (Fig. 1b). Notably, electric fields at the surface of Si blocks are enhanced by 80x. Due to the gaps between discrete silicon blocks within the resonator, 29% of the electric field energy is exposed to the surrounding medium compared with 8% in a continuous or partially notched waveguide (Supplementary Note 2 and Supplementary Fig. 4). This field concentration in the gaps leads to greater sensitivity to surface-bound analytes. Additionally, these silicon resonators exhibit sharp scattering responses in the far-field. As seen in Figure 1c, calculated reflection spectra Q-factors exceed 5,000 for Δw=50 nm, and can be further increased with decreased Δd (*vide infra*). The high-Q resonances are designed around near infrared wavelengths of 1500–1550 nm to mitigate intrinsic optical absorption due to crystalline silicon and to leverage telecom C-band infrastructure.

We fabricate silicon resonators atop a sapphire substrate (Fig. 1d) (see Methods). Utilizing a near-infrared supercontinuum laser and spectrometer equipped reflection microscope (Supplementary Fig. 1), we illuminate the metasurfaces at normal incidence and simultaneously measure the transmitted spectra from multiple resonators (Fig. 1e). By modulating the block lengths in adjacent nanostructures by ± 5 nm, we intentionally vary the spectral position of the resonant mode, highlighting that each waveguide structure can be individually addressed and tuned as a distinct resonator (Fig. 1e & 1f). Our high-Q resonances do not rely on inter-chain coupling or an extended 2-D array effect. This spatial localization of the optical modes makes our platform ideally suited for the integration of densely distributed and multiplexed sensor arrays.

## Guided-mode resonant metasurface characterization

**Fig. 2.**
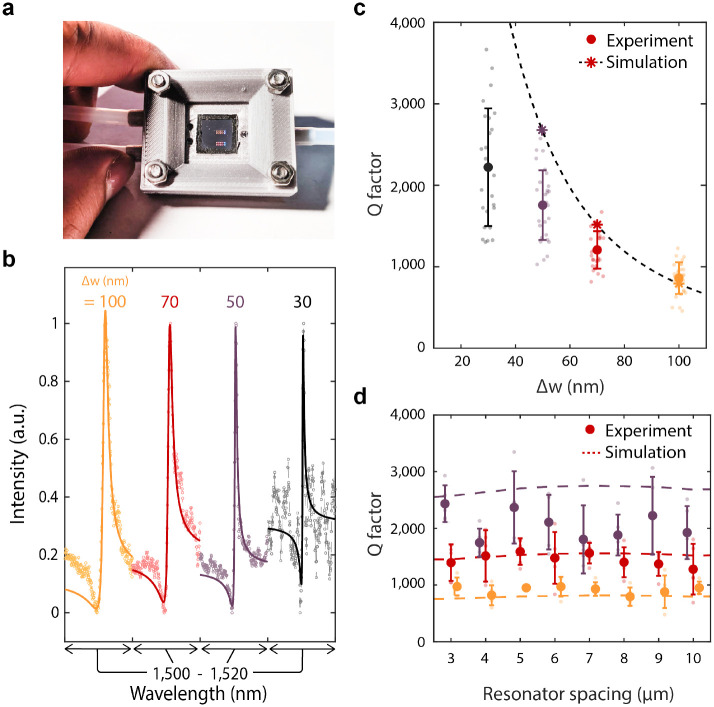
Fluid cell characterization of metasurfaces. **a,** Photo of metasurface chip enclosed in fluid cell. **b**, Representative spectra from resonators with varying Δw. Solid lines represent fits to a Lorentzian oscillator. **c**, Quality factor of resonances with different Δw. Bold markers and error bars are the mean and standard deviation for N=30 resonators at each condition. Stars represent simulated values and the dashed line is a fit to predicted values from coupled mode theory (Supplementary Note 3). **d**, Quality factor as a function resonator spacing where mean and standard deviation are for N=5 resonators at each condition.

Our metasurfaces are sealed in a 3-D printed fluid cell (Fig. 2a) and characterized in phosphate-buffered saline (PBS) solution (1x concentration) to represent physiological conditions for bio-molecule detection. In Fig. 2b, we vary the perturbation Δw along the block chain from Δw = 100 nm to Δw = 30 nm and observe a decrease in the resonant linewidth for at least 25 individual resonators at each condition (Fig. 2b & 2c). Importantly, in our high-Q metasurface design, the coupling strength between free space radiation and the GMR is dictated by the degree of asymmetry along the waveguide. As silicon is lossless in the near infrared, radiative loss dominates the GMR resonant lifetime and Q factor. Thus, by shrinking Δw we observe scattering responses with average Q factors of 800 (at Δw = 100 nm) increasing to 2,200 at Δw= 30 nm and even observing Q’s above 3,000 for individual resonators (Fig. 2c).

The observed Q factors represent a two to three order of magnitude increase compared to reported plasmonic biosensors, and a significant (>5–10x) increase compared to other non-local metasurface biosensors[22, 23, 25, 40, 41], yielding a FOM of ~400 (Supplementary Fig. 7). Our experimental Q factors are slightly lower than numerically predicted likely limited due to scattering losses caused by fabrication imperfections. We also note that water has non-negligible absorption in the 1,500 nm wavelength range that may limit our attainable experimental Q factors (Supplementary Note 3 and Supplementary Fig. 5). Designing future resonators in an optical transparency window of biological media (such as 1,300 nm) and optimizing fabrication processes may further improve performance, with Q factors in the millions potentially attainable; such structures could offer the single particle sensitivity of high-Q microcavities,[30, 32] but with the ease of integration and compact form-factor afforded by free-space coupling.

Due to the localization of the mode along each individual chain, resonators can be spaced laterally at least as close as 3 *μ*m without affecting the GMR (Fig 2d). Based on our fabricated waveguide length of 200 *μ*m, our devices feature sensor arrays with densities of over 160,000 sensors per cm^2^. Due to the slow group velocities of the GMR’s, losses due to finite size effects can be suppressed[39, 42], and 50 *μ*m waveguides can be fabricated with comparable Q (Supplementary Fig. 6), yielding feature densities over 600,000 sensors per cm^2^. These large sensor densities offer an avenue for robust statistical analysis in diagnostic studies as well as a platform for multiplexed detection of many distinct biomarkers in parallel.

## Self-assembled monolayer functionalization and sensing

**Fig. 3.**
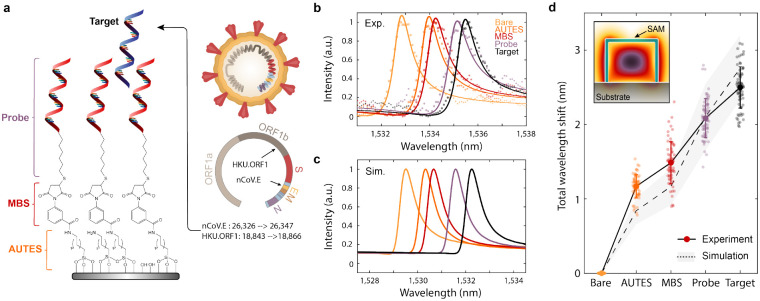
DNA monolayer functionalization and resonant wavelength shift measurement. **a**, Schematic of chemical components utilized in immobilizing DNA self-assembled monolayers (SAM) onto the silicon nanostructures. Target DNA fragments for this study are portions of the E and ORF1b genes from the SARS-CoV-2 virus. **b**, Experimentally measured and **c**, simulated resonance wavelength shift responses with the addition of each molecular layer in the SAM, including complementary nCoV.E target binding. Markers in **b**, correspond to measured data points while solid lines show fits to a Lorentzian oscillator. The difference in absolute wavelength values between experimental and simulated spectra can be attributed to slight dimension variations in the fabricated structures. **d**, Total resonant wavelength shift during SAM functionalization and DNA sensing as referenced from initial measurements on bare silicon structures. Markers represent individual measurements from N=75 independent resonator devices and bolded markers and error bars are the mean and standard deviation of the measurements.

To utilize our sensor arrays for nucleic acid detection, we modified the silicon surface with DNA monolayers, where complementary nucleic acid sequences serve as capture molecules for a specified target genetic sequence. Self-assembled monolayers (SAMs) are deposited in a three-step process to covalently link 22 base pair single-stranded DNA (ssDNA) probes over the entire metasurface chip surface. The silicon surface is first functionalized with an amine-terminated silane (11-aminoundecyltriethoxysilane, AUTES), and then cross-linked via a heterobifunctional molecule (3-maleimidobenzoic acid N-hydroxysuccinimide ester, MBS) to thiolated ssDNA probes (Methods and Supplementary Fig. 2). In this study, we considered nucleic acid fragment targets of the envelope (E) and open reading frame 1b (ORF1b) genes of the SARS-CoV-2 virus (GenBank accession: MT123293.2 positions 26326→ 26347 and 18843→ 18866, respectively, also see Supplementary Table 1)[43, 44](Fig. 3a). As a proof of principle, we use synthetic DNA targets, but note that viral RNA will analogously hybridize to complementary DNA probes[45, 46].

In Fig. 3b, measured spectra show clear shifts to the resonant wavelength as consecutive molecular monolayers of AUTES, MBS, and the probe DNA are grafted to the resonator surface. In numerical simulations, monolayers were modeled as thin dielectric shells surrounding the silicon blocks and simulated responses show close agreement with the experimental resonance shifts (Fig. 3c)(Supplementary Note 6 and Supplementary Fig. 8). Upon adding a solution of target SARS-CoV-2 gene fragments, a clear resonant shift is observed (Fig 3d). Data was collected from N=75 individual resonators. The high density of sensing elements on our chips can enable significant increases in measurement throughput compared to typical photonic sensors where signals are averaged over larger 2-D arrays. The deviation between experimental and simulated wavelength shifts for the AUTES and MBS layers is likely due to the tendency for aminosilane molecules to form multilayer structures; differences in the attachment of DNA probes and subsequent target hybridization are likely due to a strong influence of steric hindrance and electrostatic repulsion effects on the packing density and hybridization efficiency of the DNA strands[47, 48, 49, 50].

## Rapid and specific gene fragment detection

**Fig. 4.**
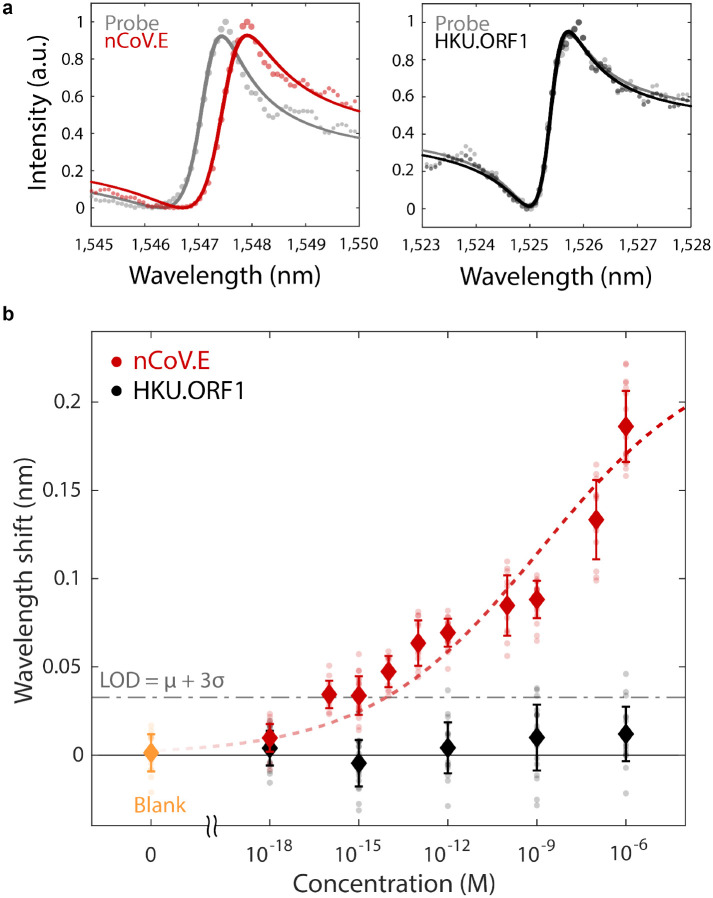
Biosensing demonstration with SARS-CoV-2 gene fragment targets. **a**, Measured spectra from individual resonators indicate significant wavelength shifts of ~0.2 nm with complementary DNA binding and minimal signal changes when introduced to non-complementary sequences. **b**, Concentration dependent binding responses for both nCoV.E and HKU.ORF1 targets incubated on metasurface devices functionalized with only nCoV.E complementary probes. Error bars indicate standard deviations of measurements from N=20 measurements from distinct resonators for each target and concentration condition. The limit of detection is estimated based on the mean + 3 standard deviations of the blank measurements. Dashed lines show fits to the Hill equation (Supplementary Note 8).

Pairing our resonators with specific probe DNA sequences offers specificity in target gene detection. We modify our surface chemical functionalization process with an antifouling matrix to reduce non-specific binding signals. A 1:1 mixture of thiolated ssDNA probes and thiolated polyethylene glycol (PEG) chains is immobilized on the silicon nanostructures, where PEG has been shown to mitigate biofouling[51, 52]. To confirm specificity, we modify target DNA strands with ATTO590 fluorescent labels and incubate sensors functionalized with probes that are only complementary to the nCoV.E sequence. Fluorescence imaging of sensors exposed to 1 *μ*M solutions of target nCoV.E and HKU.ORF1 show significant binding only for the complementary E gene target and minimal signal for the non-complementary ORF1 strands (Supplementary Fig. 10). This target specificity is also measured in the resonator scattering spectra, where resonance wavelength shifts are significant for complementary target-probe conditions and suppressed for non-specific binding (Fig. 4a).

Our sensors exhibit concentration dependent responses from 1 *μ*M to 1 aM (Fig. 4b). Measurements are taken for N=20 individual resonators at each target and concentration condition. As seen in Fig. 4b, experimental resonant shifts vary from 0.2 nm at 1 *μ*M to 0.01 nm at 1 aM. The concentration curve of nCoV.E targets is fit to the Langmuir adsorption model, which is used to describe target binding coverage in affinity-based assays (Supplementary Note 8). We estimate the limit of detection (LOD) to be ~8 fM, based on the IUPAC (International Union of Pure and Applied Chemistry) definition, which is the sum of the mean blank measurements and 3X the standard deviation of the blank measurements (LOD = *μ*+3*σ*). The LOD observed from our devices represents a significant improvement compared to previous nanophotonic nucleic acid sensor studies[15, 14, 53, 54, 55]. Furthermore, this LOD corresponds to approximately 4,000 copies/*μ*L, which is on the order of clinically measured viral loads (10^3^-10^5^ copies/*μ*L) in infected patients[56, 57]. With a detection limit in the low femtomolar regime, our sensor is promising for amplification-free and label-free viral diagnostics. We note that the target nucleic acids used in this study are only 22 base pairs in length; optimization of sensors for longer gene fragment targets could further reduce the LOD as larger molecules produce a stronger perturbation to the local refractive index. Further, the concentration dependent range of our device can potentially be tuned to different values of analyte concentration through modification of surface probe densities [8].

**Fig. 5.**
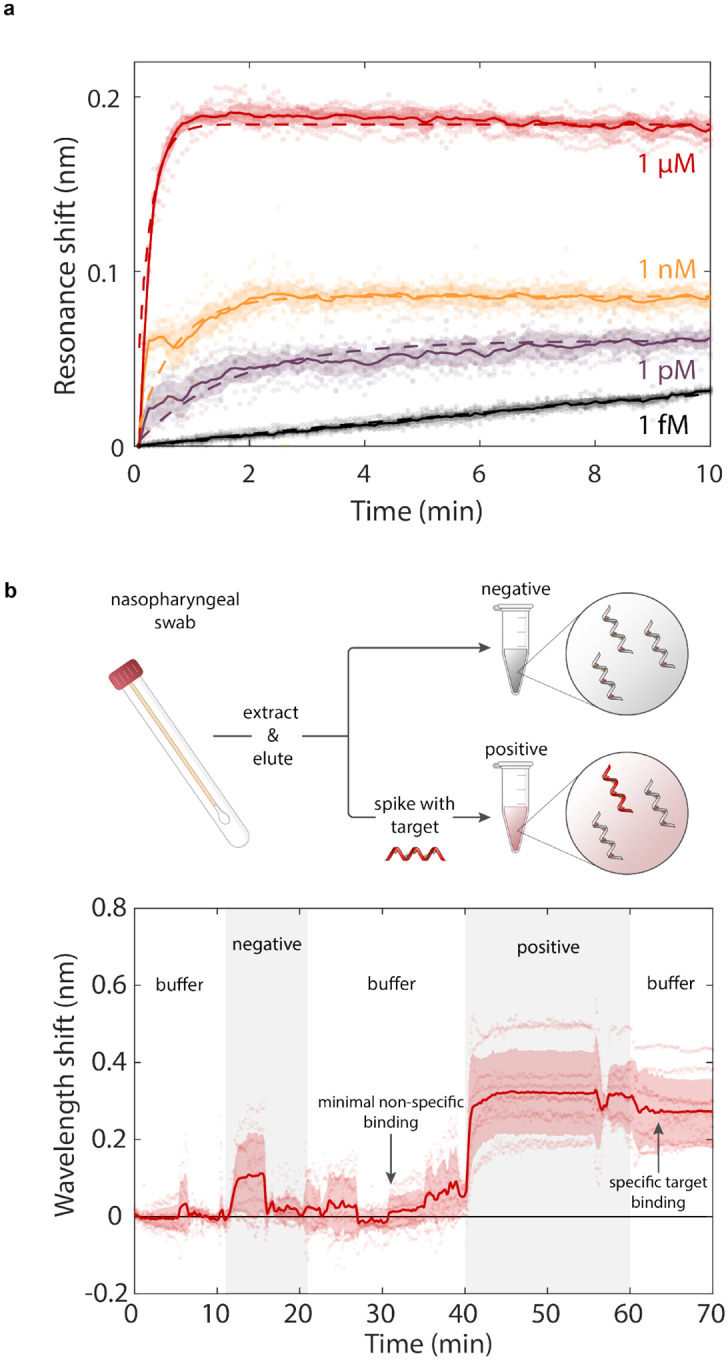
Kinetic binding response and measurement in clinical nasopharyngeal samples. **a**, Time-dependent binding responses from 10 distinct resonators exposed to 1 fM, 1pM, 1nM, and 1*μ*M concentrations of nCoV.E target molecules. **b**, Demonstration of gene fragment detection in clinical nasopharyngeal eluates. Negative samples contain random and scrambled genetic material from nasopharyngeal swabs that have been confirmed negative for SARS-CoV-2 via RT-PCR. Target nCoV.E molecules are spiked into the negative nasopharyngeal eluates at a concentration of 100 nM for the positive sample.

Efficient free-space scattering from our metasurface resonators enables real-time measurements of target binding. In Fig. 5a we show the time dependent measurement of 10 resonators with spectra acquired at 5 second intervals for concentrations of 1 *μ*M, 1nM, 1pM, and 1fM. For all concentrations, we observe resonant wavelength shifts within seconds of target injection. For 1*μ*M, 1nM, and 1pM binding curves we see signal saturation within 10 minutes as the DNA hybridization process reaches dynamic equilibrium. The signal response shows excellent agreement with the Langmuir adsorption model (dashed line Fig. 5a) where the observed hybridization rate constants of 10^−3^ to 10^−2^ s^−1^ are comparable to other hybridization capture assays[58, 59, 60]. These fast binding kinetics highlight a key advantage of chip-based approaches over conventional detection techniques that require time-intensive (~2–8 hours) molecular amplification cycles.

We further validate the performance of our metasurface sensor with clinical nasopharyngeal swabs. As a proof-of-principle demonstration, we utilize nasopharyngeal eluates from nasopharyngeal swab specimens submitted to the Stanford Clinical Virology Laboratory for SARS-CoV-2 reverse transcription polymerase chain reaction (RT-PCR) testing. The specimens were tested by a laboratory developed FDA-EUA approved RT-PCR assay targeting the E-gene. Total nucleic acid was extracted from 400 *μ*L of the swab resuspended in viral transport medium or PBS using QIAsymphony DSP Virus/Pathogen Midi Kit in QIAsymphony automated platform (Qiagen, Germantown, MD) and eluted in 60 *μ*L buffer AVE containing ~60 ng/*μ*L of carrier RNA. This study used SARS-CoV-2 negative eluates which represent sample media containing human and other nucleic acids including a high concentration milieu of poly-A or random nucleic acids of various lengths. In Figure 5b, we flow the nasopharyngeal eluates over our sensor and show that there is minimal sensor response (N=10 resonators) in negative samples, despite the background matrix of non-specific biomolecules present in clinical samples. Subsequent rinsing with pure PBS buffer returns the resonance wavelengths to baseline values. We then inject a nasopharyngeal sample solution spiked with 100 nM of complementary nCoV.E target molecules. A clear resonance wavelength shift of 0.25 nm occurs within 5 minutes; the complementary target signal remains stable as the sensor is rinsed with clean buffer solution. Therefore, our sensor is able to discriminate between specific target molecules and non-specific binding signals even in complex clinical samples, demonstrating the robustness of our self-assembled monolayer functionalized nanostructures.

## Conclusions

Our nanophotonic device offers a new platform for high throughput molecular analysis. We have demonstrated free space illuminated resonators with high-Q resonances in physiological media (2,200+) that can be patterned, tuned, and measured at densities exceeding 160,000 pixels per cm^2^. Even larger Q’s and greater feature densities can be obtained in our platform with improved fabrication processes to reduce scattering losses from structural inhomogeneities, reduced absorption losses from biological media, and inclusion of photonic mirror elements to suppress light leakage as resonator chains are truncated below 50 *μ*m. Interfaced with DNA probes, our metasurface design enables rapid, label-free, and highly digitized genetic screening that can bridge many of the challenges faced by conventional genetic analysis techniques. This increased digitization of target gene binding may also be integrated with machine learning based analysis for further improved accuracy or to allow for discrimination of small signals due to genetic variants and point mutations[61]. Paired with bioprinting procedures where different gene sequence probes are spotted across distinct sensing pixels, our high-Q metasurface chips can provide the foundation for rapid, label-free, and massively multiplexed photonic DNA microarrays. Furthermore, our nanophotonic chips are amenable to intensity imaging and/or hyperspectral imaging techniques that provide signal binding information without the need for a spectrometer[21, 40], further reducing complexity and costs towards point of care genetic screening. Our platform promises unique possibilities for widely scaled and frequently administered genetic screening for the future of precision medicine, sustainable agriculture, and environmental monitoring.

## Methods

### Computational design

Electromagnetic simulations were performed with the Lumerical FDTD Solver. Metasurfaces were simulated with periodic boundary conditions in the x and y directions and perfectly matched layer (PML) boundary conditions in the z direction. Structures were excited with a plane wave polarized at 45° and injected from the negative z direction through a sapphire substrate. Transmission spectra were computed using a power monitor placed in the far field of the metasurface in the +z direction. Cross polarized transmission intensity was calculated as Power(−45°)/(Power(−45°)+Power(+45°)).

### Fabrication

The metasurfaces were fabricated using standard lithographic procedures. First, 500 nm, single crystal silicon-on-sapphire (MTI Corp.) substrates were cleaned via sonication in acetone and isopropyl alcohol. The substrates were baked at 180 °C before spin coating with hydrogen silsesquioxane (HSQ) negative tone resist (XR-1541–06, Corning). The resist was baked for 40 min at 80 °C. To reduce charging, a charge dissipation layer (e-spacer, Showa Denko) was spin coated over the HSQ resist and baked again for 5 min at 80 °C. The metasurface patterns were defined by a 100 keV electron beam in a JEOL JBX-6300FS EBL system. Patterns were developed for 120 seconds in a 25% solution of tetramethylammonium hydroxide. Reactive ion etching with Cl2, HBr, and O2 chemistries were utilized to transfer the pattern to the silicon layer (Lam TCP 9400). The HSQ resist was removed using 2% hydrofluoric acid in water and the samples were then cleaned using a Piranha solution (9:1 H_2_SO_4_:H_2_O_2_) heated to 120 °C. The silicon nanostructures were passivated by heating for 30 min at 800 °C in a furnace to grow a ~ 4 nm oxide layer.

### Optical characterization

Resonator spectra were measured in a home-built near-infrared reflection microscope shown in Supplementary Figure 1. Samples were illuminated via a broadband NKT supercontinuum laser with a collimated fiber output. A polarizer P1 was set to create linearly polarized incident illumination at a 45° angle with respect to the metasurface structures. The illuminating beam is focused to the back focal plane of a 5X objective (Mitutoyo Plan Apochromat NIR) with a lens L1 (f=100 mm) to produce a collimated plane wave at the sample. The devices were illuminated through the sapphire substrate. Additionally, all optical measurements in this work were taken with sample chips sealed in a fluid cell and immersed in PBS 1X. The scattered light is directed through a cross-polarized polarizer P2 at −45° to reduce the substrate Fabry-Perot signal. The scattered light is then focused via a lens L3 (f=75 mm) into a SPR-2300 spectrometer (Princeton Instruments). The broadband signal is diffracted via a diffraction grating (600 g/mm, blase wavelength 600 nm, Princeton Instruments) and focused onto an air-cooled InGaAs detector (NiRvana, Princeton Instruments). Spectral features were analyzed by fitting the data with the function:

$$T={\left|a_{r}+a_{i}i+\frac{b}{f-f_{0}+\gamma i}\right|}^{2}$$

where *T* is the scattered intensity from a superposition between a constant complex background, *a*_*r*_ + *a*_*i*_*i*, and a Lorentzian oscillator with resonant frequency *f*_0_ and full-width at half-maximum of 2*γ*. The quality factor is then calculated as Q = *f*_0_/2*γ*.

### Surface functionalization

Self-assembled monolayers of single stranded probe DNA was interfaced to the silicon metasur-faces through a multi-step chemical functionalization process summarized in Supplementary Figure 2. To activate the silicon surface for functionalization, the samples were immersed in a Piranha solution (9:1 H_2_SO_4_:H_2_O_2_) heated to 120 °C for 20 min to hydroxylate the surfaces. Next, samples were immersed in a 0.1 mM solution of 11-aminoundecyltriethoxysilane (Gelest Inc.) in ethanol, sealed, and left for overnight for 18–24 hrs. The samples were rinsed in fresh ethanol for 5 min (3X) and then baked for 1 hr at 150 °C to form a stable silane layer. A hetero-bifunctional cross linking molecule was attached to the silane layer through immersion in a 1mM solution of 3-maleimidobenzoic acid N-hydroxysuccinimide ester (Millipore Sigma) dissolved in a 1:9 (v/v) mixture of dimethyl sulfoxide and PBS for 1 hr. Samples were then rinsed thoroughly with deionized water and blown dry with N_2_ gas. Single stranded DNA probes were obtained from Integrated DNA Technologies (Coralville, IA) modified with a disulfide tether on the 5’ ends. The as received DNA probes were disperesed in 50 *μ*L of tris-EDTA buffer, pH 8.0, and mixed with 30 mg of DL-dithiothreitol for at least 1 hr to reduce the disulfide moieties to thiols. The probes were then purified via gravity-flow size exclusion chromatography using illustra NAP-5 columns. The concentration of the eluted DNA solutions were determined using UV absorption signatures (Varian Cary 500 UV-Vis Spectrophotometer). For the functionalization reaction, portion of the stock solution were then diluted to 20 *μ*M in PBS 1x with added divalent cations of 100 mM MgCl_2_. For measurements presented in Fig. 4 and Fig. 5, the DNA probes were mixed in a 1:1 ratio (10*μ*M:10*μ*M) with thiolated monomethoxy polyethylene glycol (mPEG) (MW = 350) from Nanocs (Boston, MA). The DNA probe solution was pipetted onto each sample and incubated overnight (~18–24 hrs) in a dark and humid environment. Samples were rinsed with PBS 1X and then soaked in a PBS solution with added salt to a concentration of 1M NaCl for 4 hours to remove any loosely bound or physiosorbed oligonucleotides. Samples were then rinsed with PBS 1X and deionized water and dried with N_2_ gas. Samples corresponding to optical measurements in main text Fig. 3 were measured before and after each functionalization step with additional deionized water rinsing and N_2_ drying before the next chemical processing step. Samples corresponding to main text Fig. 4 adn 5 were optically characterized only before and after target DNA hybridization.

### DNA hybridization

For DNA hybridization measurements presented in Fig. 4, a baseline spectroscopic measurement was taken on metasurfaces that had been functionalized with a probe DNA monolayer. Probes with sequences corresponding to the E gene of the SARS-CoV-2 virus were used in all experiments. Following baseline measurements, samples were exposed to a solution containing either E or ORF1b gene fragments. A target DNA solution corresponding to either complementary E gene or non complementary ORF1b gene fragments (Supplementary Table 1) was produced by diluting a 100 μM stock solution to the desired concentration in 1X PBS. Additional divalent cations corresponding to 100 mM MgCl_2_ were added to the solution to increase hybridization efficiency and speed. The resonant wavelength shift for each concentration was recorded after 20 minutes of incubation.

For dynamic DNA hybridization measurements presented in Fig. 5 of the main text, samples functionalized with DNA probes were placed in a fluid cell and mounted in the optical transmission set up described above. Spectral acquisitions were collected at 5 second intervals, and measurements were conducted under a continuous flow rate of 50 *μ*L/min.

For measurements presented in Fig. 5b, nasopharyngeal swabs were collected from clinical patients at the Stanford Clinical Virology Laboratory. Nasopharyngeal swabs were submitted for SARS-CoV-2 RT-PCR testing and were tested by an FDA-EUA approved RT-PCR Assay targeting the E-gene. Total nucleic acid was extracted from 400 *μ*L of the swab resuspended in viral transport medium or PBS using QIAsymphony DSP Virus/Pathogen Midi Kit in QIAsymphony automated platform (Qiagen, Germantown, MD) and eluted in 60 *μ*L buffer AVE containing ~60 ng/*μ*L of carrier RNA. Collected samples were pooled to create the negative control sample. Synthetic E gene fragment targets were spiked into the pooled negative control samples at a concentration of 100 nM to create the positive test samples.

## Supplementary Material

1
