# Supplementary material for: Rapid genetic screening with high quality factor metasurfaces
Source: ArXiv. 2021 Oct 15:arXiv:2110.07862v2. Originally published 2021 Oct 15. Preprint. [Version 2] (PMC8528080)
Supplement: 1 [file NIHPP2110.07862V2-supplement-1.pdf]

arXiv:2110.07862v2 [physics.optics] 31 Jul 2022

Supplementary Information: Rapid genetic screening with high quality factor metasurfaces

Jack Hu[1*], Fareeha Safir[2], Kai Chang[3], Sahil Dagli[1], Halleh B. Balch[1], John M. Abendroth[4], Jefferson Dixon[2], Parivash Moradifar[1], Varun Dolia[1], Malaya K. Sahoo[5], Benjamin A. Pinsky[5,6], Stefanie S. Jeffrey[7], Mark Lawrence[8*], Jennifer A. Dionne[1*]

[1]Department of Materials Science and Engineering, Stanford University
496 Lomita Mall, Stanford, CA 94305, USA
[2]Department of Mechanical Engineering, Stanford University
440 Escondido Mall, Stanford, CA 94305, USA
[3]Department of Electrical Engineering, Stanford University
350 Jane Stanford Way, Stanford, CA 94305, USA
[4]Laboratory for Solid State Physics, ETH Zürich
CH-8093 Zürich, Switzerland
[5]Department of Pathology, Stanford University School of Medicine
300 Pasteur Drive, Stanford, CA 94305, USA
[6]Department of Medicine, Division of Infectious Diseases and Geographic Medicine,
Stanford University School of Medicine
300 Pasteur Drive, Stanford, CA 94305, USA
[7]Department of Surgery, Stanford University School of Medicine
1201 Welch Road, Stanford, CA 94305, USA
[8]Department of Electrical & Systems Engineering, Washington University in St. Louis
1 Brookings Drive, St. Louis, MO 63130, USA
*To whom correspondence should be addressed; E-mail:
hujack@stanford.edu, markl@wustl.edu, jdionne@stanford.edu

Supplementary Note 1: Dispersion calculations

Considering an unperturbed silicon waveguide made from a 1-D array of subwavelength silicon blocks, we calculate the waveguide dispersion for the lowest order mode (Supplementary Fig. 3a). The waveguide mode possesses larger momentum than free-space radiation and is ”bound” and does not couple to free-space illumination. Upon introducing periodic perturbations in the length of every other silicon block along the waveguide, our unit cell spacing, a, effectively doubles, folding the first Brillouin zone in half. Now modes that were previously inaccessible to freespace illumination lie above the light line (Supplementary Fig. 3b). This band structure is maintained when the magnitude of the perturbation is changed and only the coupling strength and Q factor are modulated as discussed in the main text.

Supplementary Note 2: Spatial distribution of electric fields around resonators

The sensitivity of a resonant mode to minute changes in the local refractive index can be estimated by the fraction of electric field energy residing outside the resonator. We calculate the exposure of the mode utilized in our sensors with the following equation:

$$f_{U_E} = \frac{\int_{V_{out}} \epsilon_{out} |E|^2 dV_{out}}{\int_{V_{in}} \epsilon_{in} |E|^2 dV_{in}}$$

where ϵ_{out} and ϵ_{in} are the permittivity of the medium containing the analyte and the permittivity of the resonator and substrate, respectively. V_{out} and V_{in} represent the volumetric regions of the analyte containing medium and the portions inside the resonator or substrate that do not overlap with any bound materials or molecules. Performing this analysis on the sensor design described in the main text as well as guided mode resonant structures previously described in reference [1] composed of notched silicon waveguides, we find that our silicon block chains significantly increase field penetration into the surrounding environment. Field profiles of the two structures are plotted in Supplementary Fig. 4 showing similar transverse electric waveguide modes. Due to the subwavelength spacing of the discrete silicon blocks in our sensors, we still excite the localized waveguide modes along the periodic direction that are seen in continuous silicon wire waveguides. However, the grating-like structure exposes regions of the mode to the surroundings while also reducing the effective mode index of the waveguide, leading to further extension of the fields out of the resonator. This design results in the fraction of the mode energy in the surroundings to increase to $f_{U_E} = 0.29$ compared to only $f_{U_E} = 0.08$ for notched or continuous waveguide structures.

Supplementary Note 3: Quality factor scaling and water absorption

As discussed in the main text, introducing an asymmetry along a silicon waveguide allows for the excitation of previously bound modes. Reduction of the asymmetry, Δw in the case of our metasurfaces, decreases the coupling strength of the mode to free-space radiation thereby increasing the Q factor. For a material that exhibits no intrinsic absorption losses, such as silicon in the near infrared, the Q factor can be arbitrarily increased as the perturbation strength approaches zero. This dependence of the Q factor on subtle structural deviations have been previously described through temporal coupled-mode theory and perturbation theory[2, 3, 4]:

$$Q = \frac{B}{\alpha^2}$$

where B is a constant that depends on the resonator geometry and α is a unit-less asymmetry parameter represented by $\Delta \mathrm{w}/\mathrm{w}_0$ in our metasurface. This relationship is shown in Supplementary Fig. 5, where theory (solid line) and numerical simulations (stars) indicate diverging Q factors as Δw is decreased. We also observe that experimentally observed Q factors are lower than predicted values (experimental data from Main text Fig. 2). One significant factor limiting our experimental quality factors is the absorption coefficient of water at telecommunication wavelengths. Since all our optical measurements are performed in aqueous solutions, dissipative losses are expected to decrease our measured Q factors as shown by the dashed line in Supplementary Fig. 5, which represents numerical calculations including water absorption. The effects of absorption losses are particularly strong as Δw is decreased, as longer resonance lifetimes lead to greater interaction between the resonant mode and the absorptive background medium. Future iterations of our sensor can be designed in the water absorption window around 1300 nm to maximize performance of the resonators. Additionally, fabrication imperfections such as surface roughness or non-uniformity in the metasurface structures will introduce scattering losses and reduce the observed Q factor.

Supplementary Note 4: Finite size resonators

While the resonators shown in the main text exhibit high-Q modes in longer 1-D arrays (200 μm), we show that the resonators can be scaled down significantly while maintaining sharp spectral features. Our metasurface design features low scattering losses out the ends of the waveguides, and hence are relatively robust to resonator finite size effects due to the high index contrast between separated silicon blocks and gaps containing the background medium. In Supplementary Fig. 6a, we show calculated dispersion diagrams for three different resonators consisting of a solid silicon waveguide with increasing depths of notch corrugations. The waveguide has width of 600 nm and from top to bottom, the bands correspond to notches added on both sides of the waveguide with

depths of 50, 150, and 300 nm. We observe flattening of the bands as the notch depth is increased until 300 nm, where the waveguide is now separated into distinct silicon blocks. The flatter bands indicate a much smaller group velocity due to strong in-plane Bragg scattering, which reduces the propagation of the mode out the waveguide ends and reduces effects of shrinking the resonator on the Q factor. We experimentally verify that we can maintain high quality factors while shortening the overall length of each resonator. In Supplementary Fig. 6b,c we show SEM images of multiple resonators with varying lengths from 300 μm down to 50 μm and representative spectra. Fitting N=6 resonators for each condition, Supplementary Fig. 6d shows little change in the Q factor with varying waveguide length. Resonators with Δw = 50 and 30 nm maintain high Q factors exceeding 1000 even in resonators down to 50 μm. Each resonator could potentially be further scaled down with added dielectric mirrors patterned on the waveguide ends to reduce scattering losses. Thus, it is possible to envision individual free space coupled high Q resonators on the order of a few μm.

Supplementary Note 5: Refractive index sensing figure of merit

Affinity based sensors that rely on spectral shifts induced by environmental refractive index shifts are often evaluated by a Figure of Merit (FOM):

$$FOM = \frac{(\Delta\lambda/\Delta n) * Q}{\lambda_0}$$

where $\Delta\lambda/\Delta n$ s the resonant wavelength shift induced by a change in the background medium refractive index or bulk refractive index sensitivity, Q is the resonator quality factor, and λ_0 is the resonant wavelength. To determine the bulk refractive index sensitivity of our devices, we take a series of optical measurements in various saline solutions with differing concentrations of NaCl dissolved in deionized water. Increasing NaCl concentrations have been shown to increase the refractive index of water.[5] In Supplementary Fig.7, the change in resonant wavelength indicates our sensors have a sensitivity of $\Delta\lambda/\Delta n$ = 270 nm/RIU. The sensitivity of the resonators does not vary significantly with Δw, as the block asymmetry alters the Q factor, but the modal overlap with the surrounding medium does not change. Given our resonator Q factors of $\sim$2200 (Main text Fig. 2), we obtain a sensing FOM of around 400. This value is larger than previous demonstrations in plasmonic or dielectric metasurface based sensors.[6, 7, 8, 9, 10] We also note that modification of our metasurfaces, such as introducing subwavelength gaps or slots along the waveguide that further expose the resonant mode to target analytes, could dramatically improve the sensitivity and FOM of future iterations of the devices.

Supplementary Note 6: Modeling self-assembled monolayers

To estimate the resonant wavelength shifts corresponding to successive molecular layers deposited on our sensors (Main text Fig. 3c), we model each surface step with a thin dielectric shell and numerically calculate the response with FDTD as described above. The dielectric shells extend from all exposed silicon faces of our metasurface nanoblocks as shown in Supplementary Fig. 8. The initial bare sensor before surface functionalization is calculated with a 4 nm silicon dioxide layer due to the thermal passivation step performed after nanofabrication of our sensors.[11] The AUTES layer is assumed to bind as a uniform monolayer of thickness 1.8 nm and refractive indices ranging from n = 1.40-1.45, based on reported literature values. The refractive index is calculated for a range that corresponds to typical estimated optical properties of biomolecular layers. The MBS layer is estimated as a 0.7 nm thick layer based on the reported spacer arm length of the molecule and refractive index of n = 1.40-1.45. The thiolated ssDNA probe layers are calculated with optical properties n = 1.37-1.382, accounting for potential reduction in density due to dilution with PEG chains.[12, 13, 14] We estimate the structure of our single-stranded DNA probes using the open source software "mfold" to predict the secondary structure in a 1X PBS solution and the most stable conformation is shown in Supplementary Fig. 9. The probe layer thickness is estimated by the wormlike chain model[15]:

$$R_g^2 = \left(\frac{lL_p}{3}\right) - L_p^2 + \left(\frac{2L_p^3}{l}\right) - \left(\frac{2L_p^4}{l^2}\right)(1 - e^{-l/L_p})$$

where the monomer spacing a = 0.6 nm, persistence length L_p = 1 nm, and contour length l = Na (N = number of nucleotides) give a radius of gyration, Rg, and layer thickness of $\sim$ 4 nm.[16] The dsDNA probe refractive index is approximated as n = 1.4-1.423 as estimated from the densification and increased polarizability of duplexed DNA compared to single stranded DNA.[17] The thickness is similarly estimated by the wormlike chain model, but with a = 0.3 nm and Lp = 30 nm, which returns a similar radius of gyration of 4 nm. Based on the short fragment length of our probe and targets at 22-26 nt, we do not expect a significant monolayer thickness change upon DNA hybridization.

Supplementary Note 7: Fluorescence microscopy

Fluorescence experiments were performed after DNA hybridization experiments with target nucleic acids tagged with ATTO590 dye on the 5' end. Dried samples were placed in a Zeiss AxioImager system and imaged with a 20x objective. Fluorescence images were acquired with 1000 ms exposures on a Zeiss Axiocam 506 mono camera. Fluorescence intensity values were averaged over a 80 x 40 μm area and were normalized to the maximum intensity values from chips hybridized with complementary E gene targets as seen in Supplementary Fig. 10.

Supplementary Note 8: Langmuir adsorption model

Concentration dependent resonant wavelength shift responses in main text Fig. 4 were fit to the Hill equation (formally equivalent to the Langmuir isotherm):

$$\theta = \frac{\theta_{max} * X^h}{K_d^h + X^h}$$

where θ_{max} is the saturated maximum binding signal at high target concentrations, X is the target concentration, h is the Hill coefficient which describes the slope of the curve, and K_d is the concentration value that corresponds to half-maximum binding signals. Furthermore, the Langmuir adsorption model is also used to fit the time varying responses in main text Fig. 5. The wavelength shift response as a function of time is described as[18]:

$$\theta(t) = \theta_{eq}(1 - e^{-kt})$$

where θ_{eq} is the saturated equilibrium binding signal and k is an ”observed” rate constant that accounts for both target hybridization and reversible dehybridization rates.

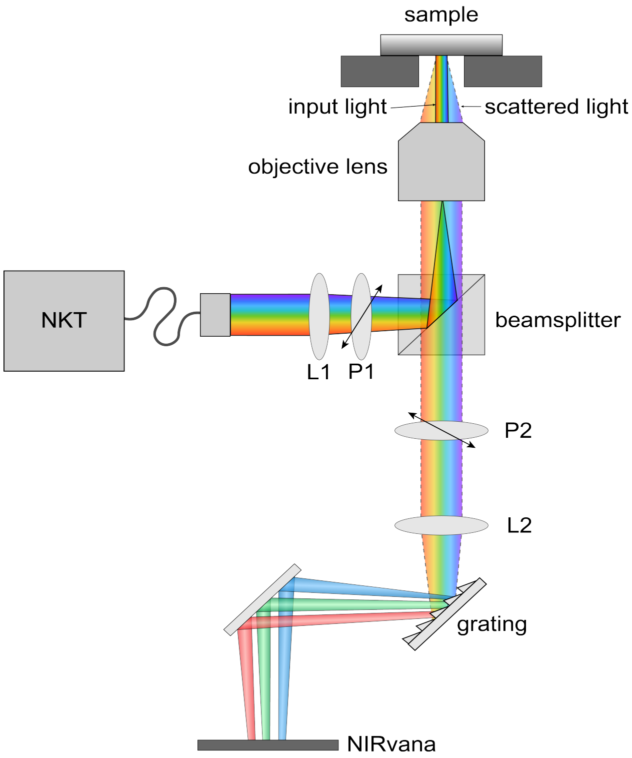

Supplementary Fig. 1. Schematic of near-infrared microscope set up utilized to collect spectra from metasurface samples.

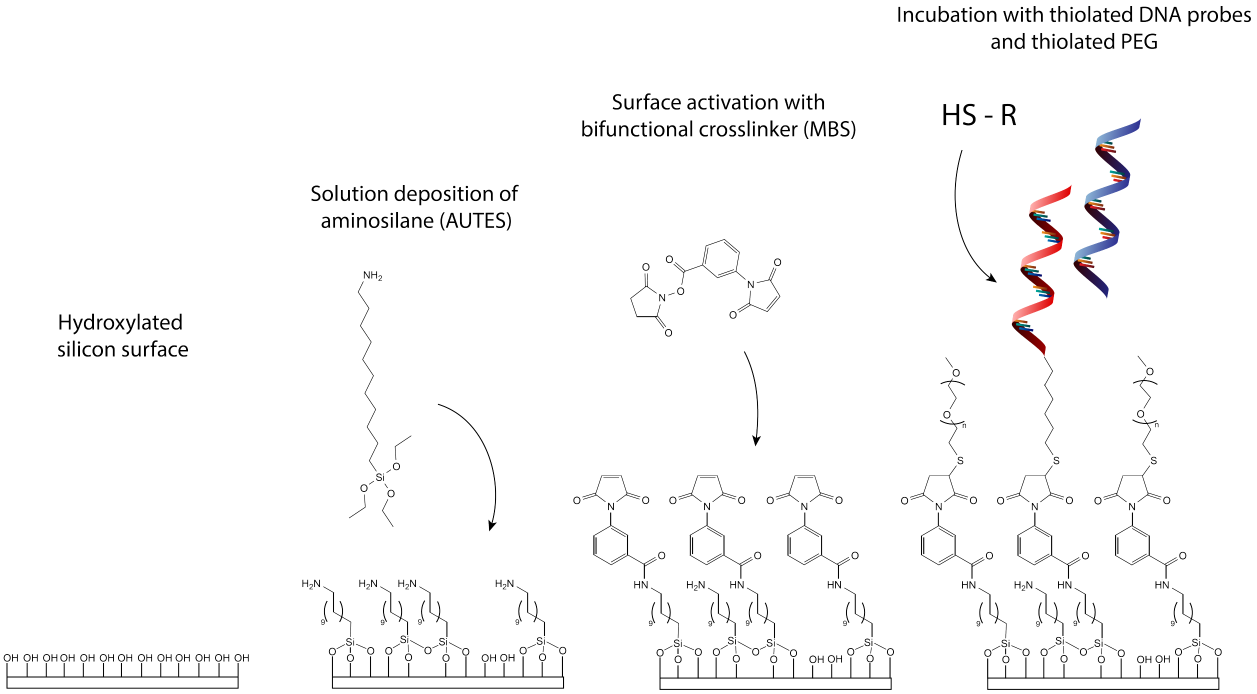

Supplementary Fig. 2. Multi-step surface functionalization of a DNA probe monolayer on silicon metasurfaces.

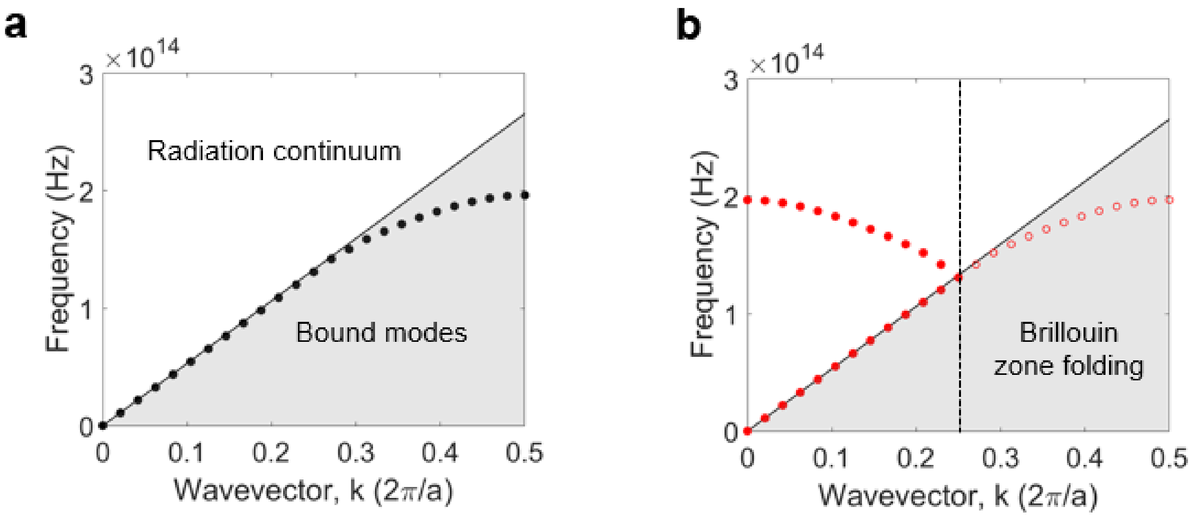

Supplementary Fig. 3. **a**, Simulated waveguide dispersion in an unperturbed chain of subwavelength silicon blocks. **b**, Brillouin zone folding introduced via symmetry breaking in biperiodic guided mode resonator.

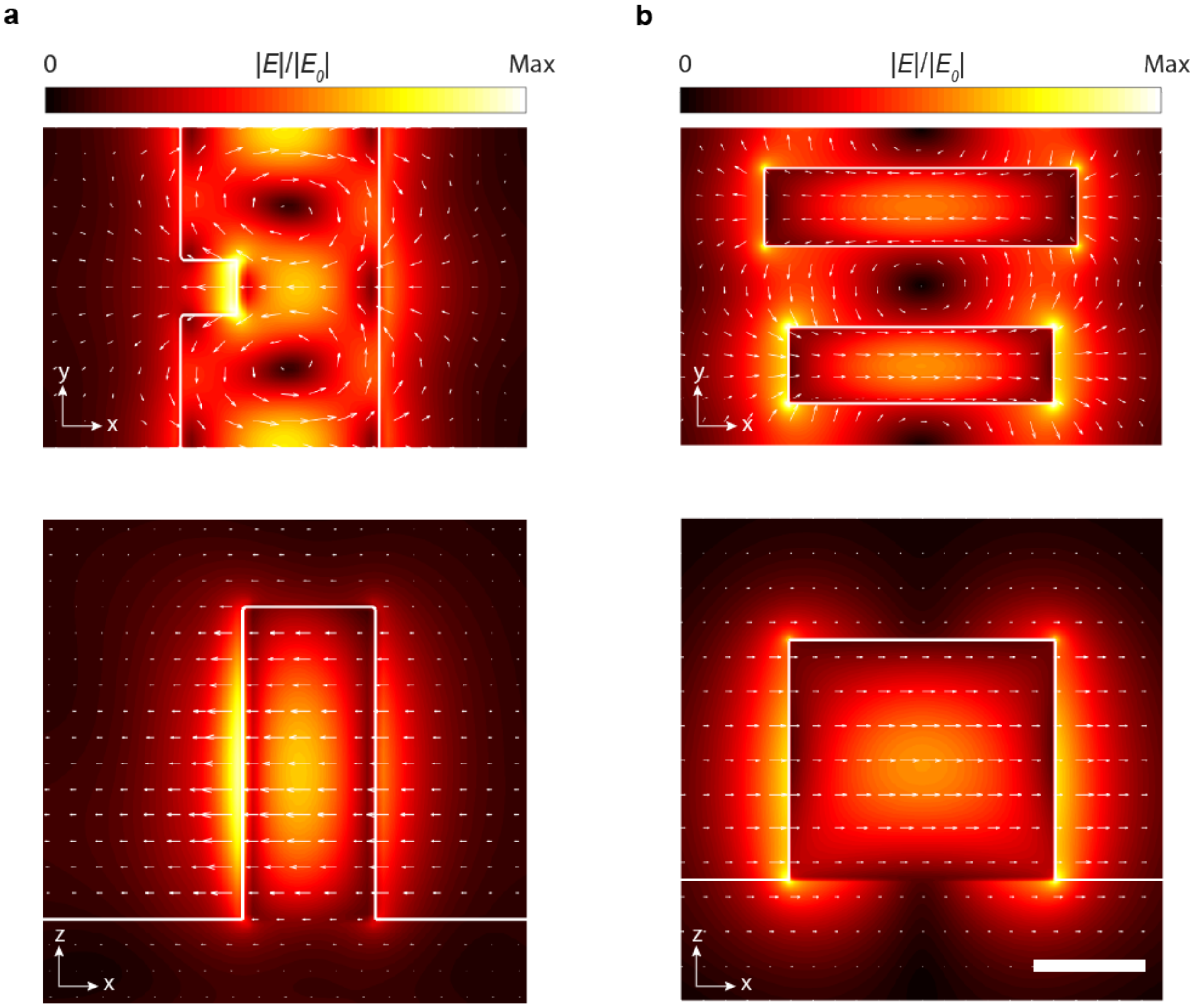

Supplementary Fig. 4. **a**, Electric field profile for notched silicon waveguide on a sapphire substrate. Upper panel shows an x-y cut through the center of the structure and the lower panel is an x-z cut through the center of the notch perturbation where fields are most strongly concentrated. **b**, Electric field profile for asymmetric chain of silicon blocks on a sapphire substrate. Upper panel represents the x-y cut through the center of the structure and the lower panel is an x-z cut through the center of the smaller block. Scale bar is 200 nm.

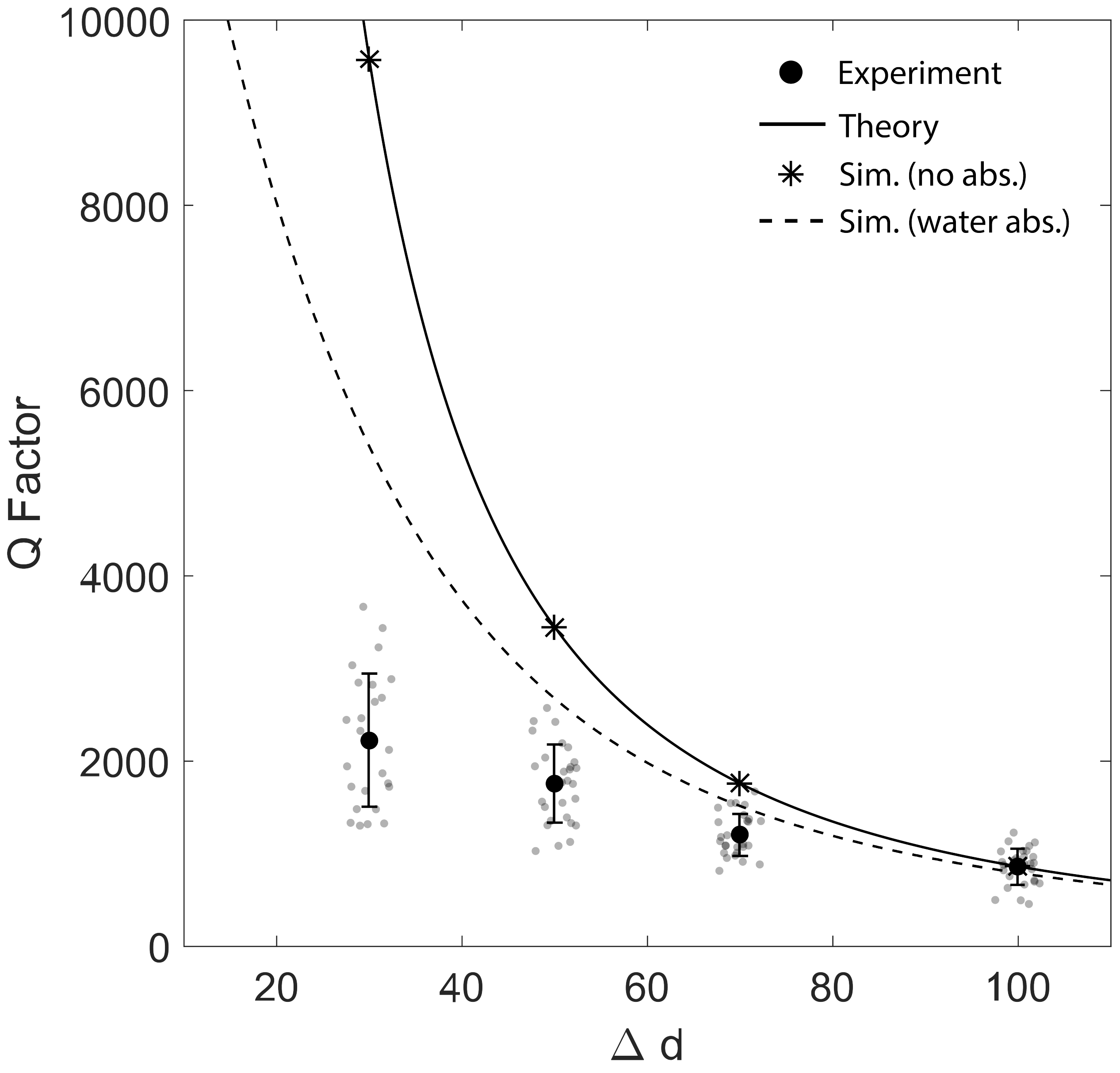

Supplementary Fig. 5. Resonator quality factor as a function of difference in neighboring silicon block length, Δd.

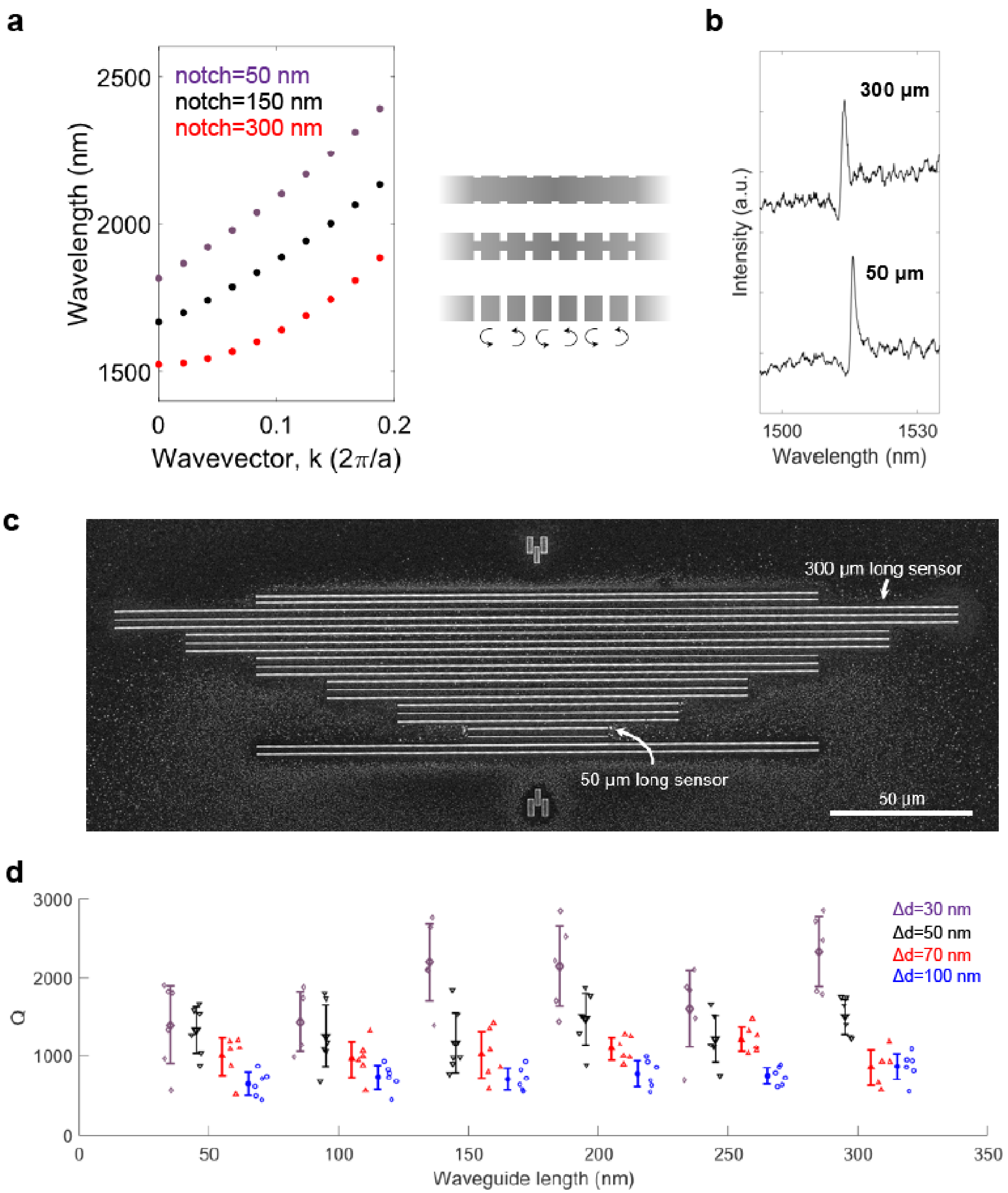

Supplementary Fig. 6. **a**, Simulated waveguide dispersion in waveguides with varying notch depth. **b**,Representative optical spectra from waveguides with length of 300 μm and 50 μm. **c**, SEM image of resonators fabricated with different waveguide lengths. **d**, Quality factors as a function of waveguide length.

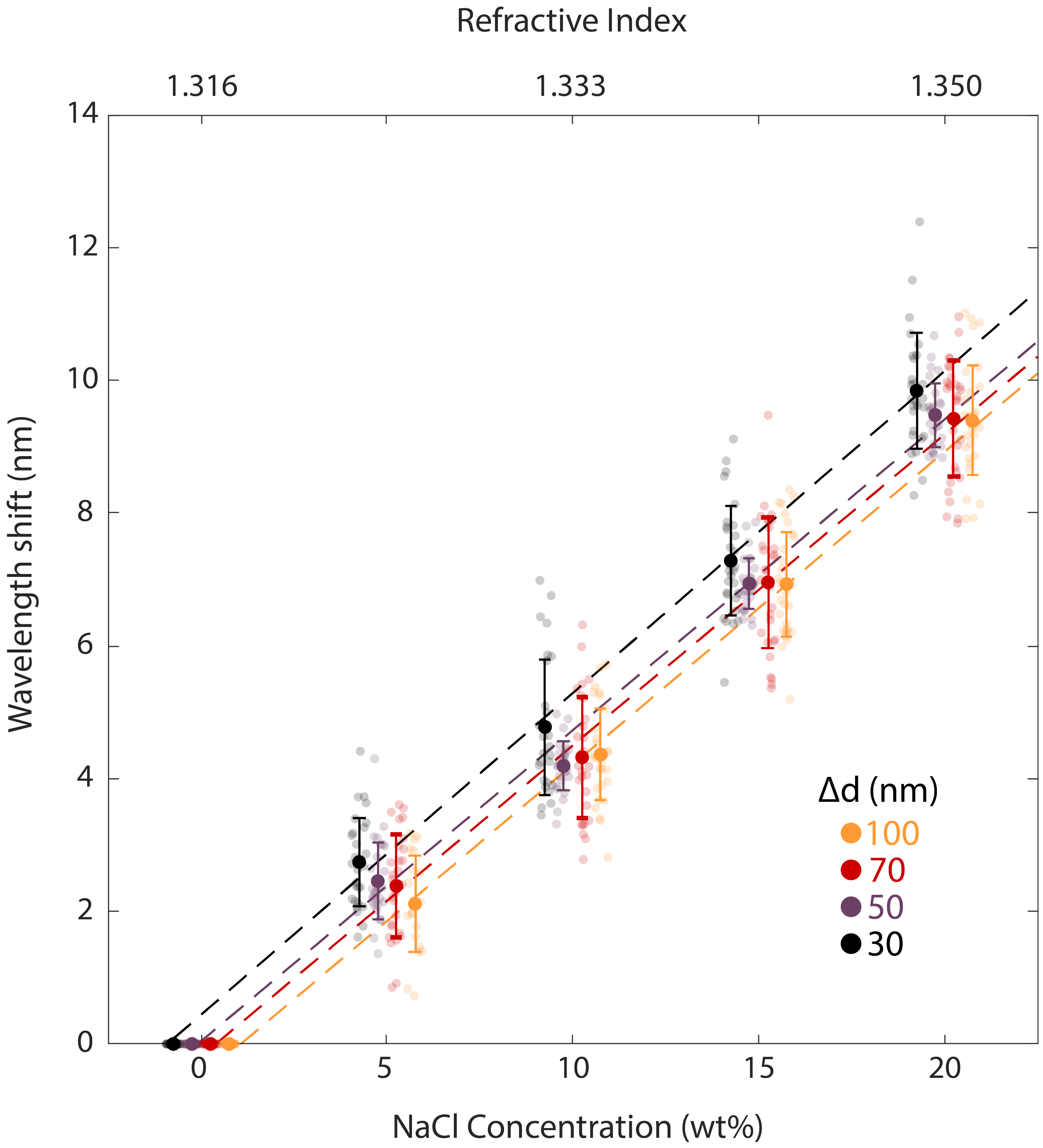

Supplementary Fig. 7. Resonant wavelength measurements as a function of background medium refractive index. Circles and error bars represent experimental measurements for N=25-30 resonators at each condition. Dashed lines represent linear fits to the data.

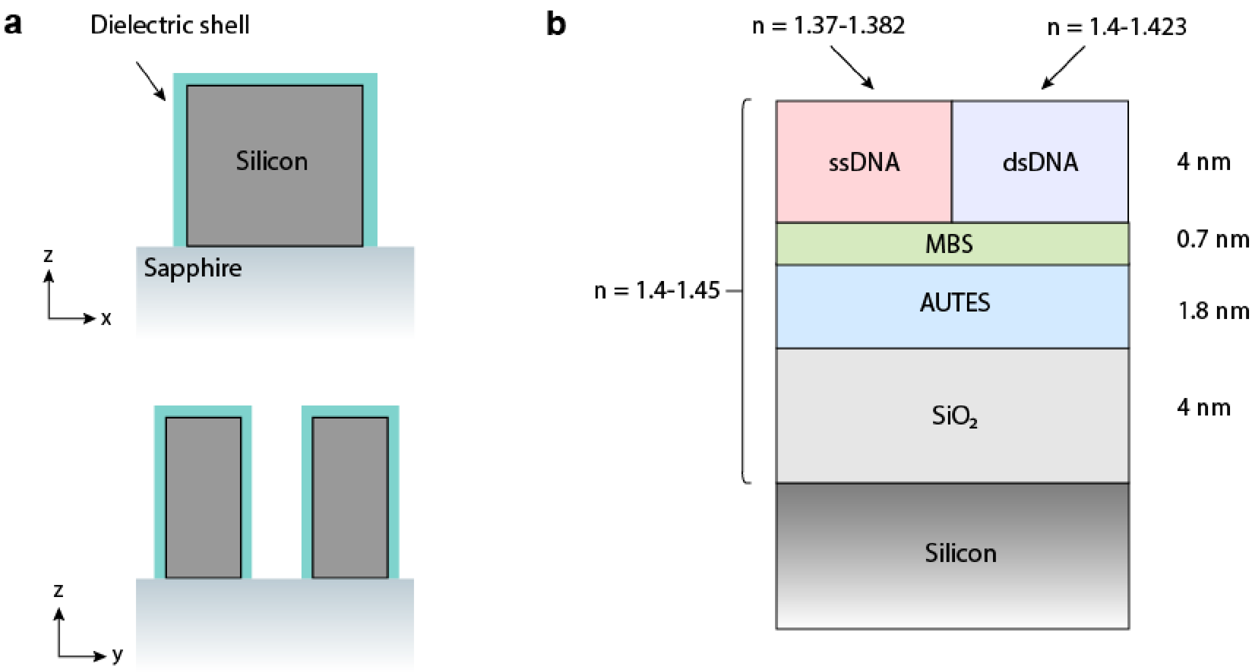

Supplementary Fig. 8. **a**, Schematic of calculated structure with dielectric shell around silicon nanostructures representing the molecular monolayers. **b**, Estimated layer thicknesses and refractive indices.

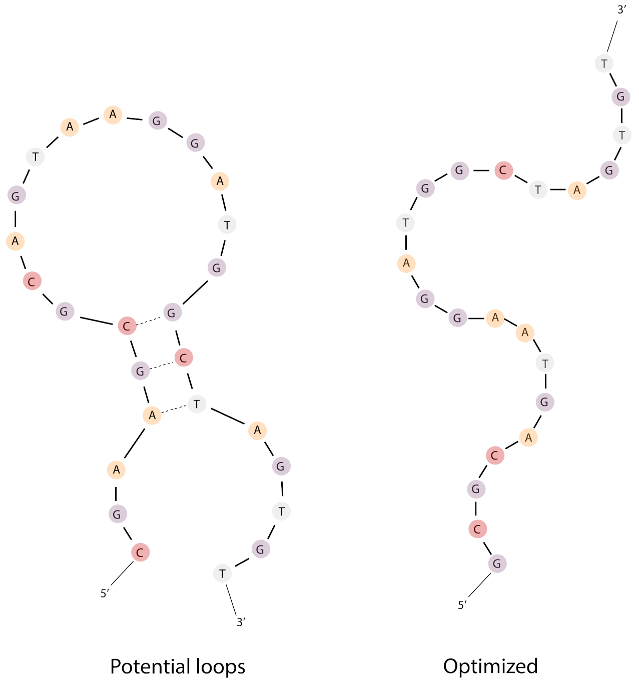

Supplementary Fig. 9. Likely secondary structure of DNA probes used in this work. Probes sequences were optimized to avoid formation of stable loop structures.

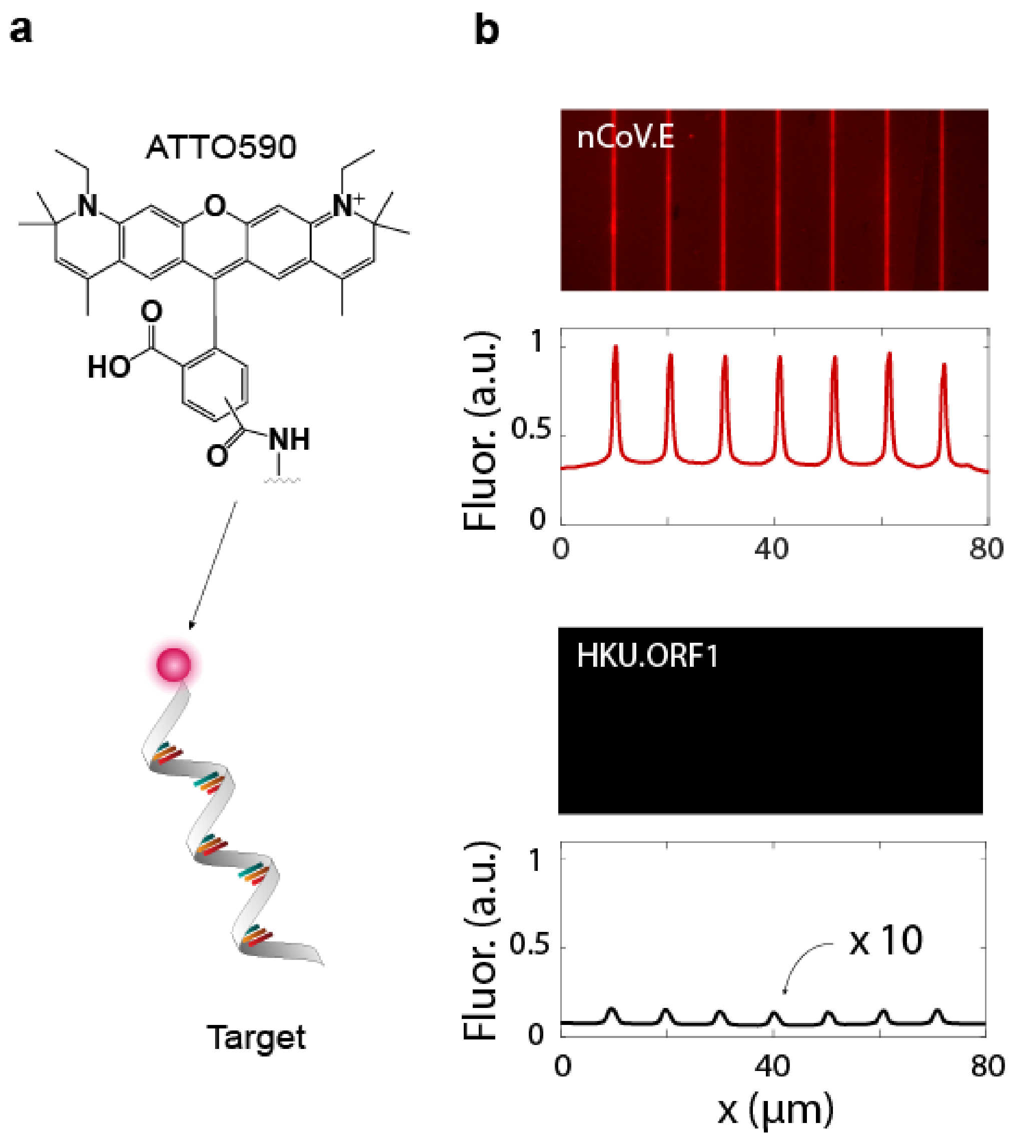

Supplementary Fig. 10. **a**, Schematic of fluorescently tagged target DNA sequences. **b**, Fluorescence images and integrated intensities for sensors exposed to complementary nCoV.E sequences (top) and non-complementary HKU.ORF1 sequences (bottom). Fluorescence imaging confirms the specificity of immobilized DNA probe molecules to complementary nucleic acid sequences. All metasurface sensors were functionalized with probes complementary only to the nCoV.E sequence.

E gene Probe	5’ - G CGC AGT AAG GAT GGC TAG TGT - 3’
E gene Target	5’ - ACA CTA GCC ATC CTT ACT GCG C - 3’
ORF1b gene Target	5’ - CT AGT CAT GAT TGC ATC ACA ACT A - 3’

Supplementary Table 1. Probe and target sequences used in this work.

References

[1] Lawrence, M. et al. High quality factor phase gradient metasurfaces. Nature Nanotechnology **15**, 956–961 (2020).

[2] Fan, S. & Joannopoulos, J. D. Analysis of guided resonances in photonic crystal slabs. Physical Review B **65** (2002).

[3] Overvig, A. C., Shrestha, S. & Yu, N. Dimerized high contrast gratings. Nanophotonics **7**, 1157–1168 (2018).

[4] Koshelev, K., Lepeshov, S., Liu, M., Bogdanov, A. & Kivshar, Y. Asymmetric metasurfaces with high-q resonances governed by bound states in the continuum. Physical Review Letters **121**, 193903 (2018).

[5] Saunders, J. E., Sanders, C., Chen, H. & Loock, H.-P. Refractive indices of common solvents and solutions at 1550 nm. Applied Optics **55**, 947–953 (2016).

[6] Conteduca, D. et al. Dielectric nanohole array metasurface for high-resolution near-field sensing and imaging. Nature Communications **12**, 3293 (2021).

[7] Yang, Y., Kravchenko, I. I., Briggs, D. P. & Valentine, J. All-dielectric metasurface analogue of electromagnetically induced transparency. Nature Communications **5**, 5753 (2014).

[8] Wang, J. et al. All-dielectric crescent metasurface sensor driven by bound states in the continuum. Advanced Functional Materials 2104652 (2021).

[9] Yesilkoy, F. et al. Ultrasensitive hyperspectral imaging and biodetection enabled by dielectric metasurfaces. Nature Photonics **13**, 390–396 (2019).

[10] Triggs, G. J. et al. Chirped guided-mode resonance biosensor. Optica **4**, 229–234 (2017).

[11] Krzeminski, C., Larrieu, G., Penaud, J., Lampin, E. & Dubois, E. Silicon dry oxidation kinetics at low temperature in the nanometric range: Modeling and experiment. Journal of Applied Physics, American Institute of Physics **101**, 064908–1–8 (2007).

[12] Schreck, J. S. et al. Dna hairpins destabilize duplexes primarily by promoting melting rather than by inhibiting hybridization. Nucleic Acids Research **43**, 6181–6190 (2015).

[13] Gao, Y., Wolf, L. K. & Georgiadis, R. M. Secondary structure effects on dna hybridization kinetics: a solution versus surface comparison. Nucleic Acids Research **34**, 3370–3377 (2006).

[14] Pinto, G., Canepa, P., Canale, C., Canepa, M. & Cavalleri, O. Morphological and mechanical characterization of dna sams combining nanolithography with afm and optical methods. Materials **13**, 2888 (2020).

[15] Sim, A. Y., Lipfert, J., Herschlag, D. & Doniach, S. Salt dependence of the radius of gyration and flexibility of single-stranded dna in solution probed by small-angle x-ray scattering. Physical Review E **86**, 021901 (2012).

[16] Mantelli, S., Muller, P., Harlepp, S. & Maaloum, M. Conformational analysis and estimation of the persistence length of dna using atomic force microscopy in solution. Soft Matter **7**, 3412 (2011).

[17] Elhadj, S., Singh, G. & Saraf, R. F. Optical properties of an immobilized dna monolayer from 255 to 700 nm. Langmuir **20**, 5539–5543 (2004).

[18] Su, Q., Vogt, S. & Nöll, G. Langmuir analysis of the binding affinity and kinetics for surface tethered duplex dna and a ligand-apoprotein complex. Langmuir **34**, 14738–14748 (2018).
